# The chromosome-scale genome assembly for the West Nile vector *Culex quinquefasciatus* uncovers patterns of genome evolution in mosquitoes

**DOI:** 10.1186/s12915-024-01825-0

**Published:** 2024-01-25

**Authors:** Sergei S. Ryazansky, Chujia Chen, Mark Potters, Anastasia N. Naumenko, Varvara Lukyanchikova, Reem A. Masri, Ilya I. Brusentsov, Dmitriy A. Karagodin, Andrey A. Yurchenko, Vitor L. dos Anjos, Yuki Haba, Noah H. Rose, Jinna Hoffman, Rong Guo, Theresa Menna, Melissa Kelley, Emily Ferrill, Karen E. Schultz, Yumin Qi, Atashi Sharma, Stéphane Deschamps, Victor Llaca, Chunhong Mao, Terence D. Murphy, Elina M. Baricheva, Scott Emrich, Megan L. Fritz, Joshua B. Benoit, Igor V. Sharakhov, Carolyn S. McBride, Zhijian Tu, Maria V. Sharakhova

**Affiliations:** 1 Department of Entomology, Virginia Polytechnic and State University, Blacksburg, VA USA; 2 Department of Molecular Genetics of Cell, NRC “Kurchatov Institute”, Moscow, Russia; 3 Genetics, Bioinformatics, Computational Biology Program, Virginia Polytechnic and State University, Blacksburg, VA USA; 4 Department of Biochemistry, Virginia Polytechnic and State University, Blacksburg, USA; 5 Department of Entomology, University of Maryland, College Park, MD USA; 6 Group of Genomic Mechanisms of Development, Institute of Cytology and Genetics, Novosibirsk, Russia; 7 Laboratory of Structural and Functional Genomics, Novosibirsk State University, Novosibirsk, Russia; 8 Laboratory of Cell Differentiation Mechanisms, Institute of Cytology and Genetics, Novosibirsk, Russia; 9 Department of Ecology and Evolutionary Biology, Princeton University, Princeton, NJ USA; 10 National Center for Biotechnology Information, National Library of Medicine, National Institutes of Health, Bethesda, MD 20894 USA; 11 Department of Biological Sciences, University of Cincinnati, Cincinnati, OH USA; 12 County of San Diego Vector Control Program, San Diego, CA USA; 13 Mosquito and Vector Management District of Santa Barbara County, Santa Barbara, CA USA; 14 Corteva Agriscience, Johnston, IA USA; 15 Biocomplexity Institute & Initiative University of Virginia, Charlottesville, VA USA; 16 Department of Electrical Engineering & Computer Science, the University of Tennessee, Knoxville, TN USA; 17 Fralin Life Sciences Institute, Virginia Polytechnic and State University, Blacksburg, VA USA; 18 Department of Genetics and Cell Biology, Tomsk State University, Tomsk, Russia; 19 Princeton Neuroscience Institute, Princeton University, Princeton, NJ USA

**Keywords:** Mosquito, Genome assembly, Annotation, Genome evolution

## Abstract

**Background:**

Understanding genome organization and evolution is important for species involved in transmission of human diseases, such as mosquitoes. Anophelinae and Culicinae subfamilies of mosquitoes show striking differences in genome sizes, sex chromosome arrangements, behavior, and ability to transmit pathogens. However, the genomic basis of these differences is not fully understood.

**Methods:**

In this study, we used a combination of advanced genome technologies such as Oxford Nanopore Technology sequencing, Hi-C scaffolding, Bionano, and cytogenetic mapping to develop an improved chromosome-scale genome assembly for the West Nile vector *Culex quinquefasciatus*.

**Results:**

We then used this assembly to annotate odorant receptors, odorant binding proteins, and transposable elements. A genomic region containing male-specific sequences on chromosome 1 and a polymorphic inversion on chromosome 3 were identified in the *Cx. quinquefasciatus* genome. In addition, the genome of *Cx. quinquefasciatus* was compared with the genomes of other mosquitoes such as malaria vectors *An. coluzzi* and *An. albimanus*, and the vector of arboviruses *Ae. aegypti*. Our work confirms significant expansion of the two chemosensory gene families in *Cx. quinquefasciatus*, as well as a significant increase and relocation of the transposable elements in both *Cx. quinquefasciatus* and *Ae. aegypti* relative to the Anophelines. Phylogenetic analysis clarifies the divergence time between the mosquito species. Our study provides new insights into chromosomal evolution in mosquitoes and finds that the X chromosome of Anophelinae and the sex-determining chromosome 1 of Culicinae have a significantly higher rate of evolution than autosomes.

**Conclusion:**

The improved *Cx. quinquefasciatus* genome assembly uncovered new details of mosquito genome evolution and has the potential to speed up the development of novel vector control strategies.

**Supplementary Information:**

The online version contains supplementary material available at 10.1186/s12915-024-01825-0.

## Background

Mosquitoes pose serious health problems for humans serving as vectors for various pathogens including malarial parasites, dengue, Zika, West Nile viruses, and filarial worms [1, 2]. Global climate change, increased urbanization, travel, trade, and other human activities raise a concern about re-emergence and expansion of mosquito-borne diseases [3]. In response to this threat, novel genome-based strategies have been proposed to significantly improve existing tools for mosquito control [4]. Thus, the number of sequenced mosquito genomes available through public databases is rapidly increasing. Progress in advanced genome technologies, such as long-read technologies from Pacific Biosciences (PacBio) [5], and Oxford Nanopore Technology (ONT) sequencing [6, 7], Hi-C scaffolding [8, 9], and optical mapping [10] have enabled the development of new, high-quality, chromosome-scale assemblies [11]. Nevertheless, many mosquito genomes are still far from complete, containing hundreds of relatively short scaffolds with low overall continuity. Before our study, one of such species was the West Nile [12] vector *Culex quinquefasciatus.*

Most of the mosquitoes with chromosome-level assemblies belong to the subfamily Anophelinae [13–20]. Due to the large genome sizes and lack of good-quality polytene chromosomes, the development of chromosome-scale assemblies for mosquitoes from the Culicinae subfamily remains challenging. Only three chromosome-scale genome assemblies, assembled to the chromosome level, has been constructed for a species in this subfamily: the genome of *Aedes aegypti* [21], and recently published genomes of *Cx. pipiens molestus* and *Cx. p. palens* [22]. However, the recent genome assembly attempts for *Cx. tarsalis* [23] and improved versions of the *Ae. albopictus* genome [24, 25] did not generate chromosome-scale assemblies.

The original sequencing of the genomes of *Anopheles gambiae* [26], *Ae. aegypti* [27], and *Cx. quinquefasciatus* [28] revealed some important insights into mosquito evolution. Mosquitoes diverged from other dipterans approximately 250 million years ago (MYA). A major split in the mosquito family occurred about 150 MYA, giving rise to two subfamilies: malaria mosquitoes Anophelinae and vectors of arboviruses Culicinae. The Culicinae subfamily further diverged into two tribes, Culicini and Aedini. Interestingly, the genomes of some Culicines have increased up to 5 times in size due to dramatic expansions of transposable elements and other repeats. Initial studies showed that the *Cx. quinquefasciatus* genome size of 579 Mb [28] is about 2-fold larger than the 270 Mb genome of the malaria vector *An. gambiae* [26]. In contrast, the *Cx. quinquefasciatus* genome is about 2-fold smaller than the 1376 Mb genome of the dengue vector *Ae. aegypti* [27]. Repeat contents were originally estimated at 16, 29, and 50% in the genomes of *An. gambiae*, *Cx. quinquefasciatus*, and *Ae. aegypti*, respectively [28]. Later estimates based on chromosome-scale assembly increased the percentage of transposable elements (TEs) in the *Ae. aegypti* genome to 54.9% [21].

Comparison of the first-generation mosquito genome assemblies suggested significant gene-family expansions in *Cx. quinquefasciatus*, including olfactory and gustatory receptors, salivary gland genes, and genes associated with xenobiotic detoxification [28] compared to *An. gambiae* [26] and *Ae. aegypti* [27]. However, only ~9% of the original *Cx. quinquefasciatus* genome was assigned to chromosomes. Due to high fragmentation of the genome, subsequent use of classical physical and genetic mapping insignificantly increased the mapped portion of the genome to 13% [29]. Although the original *Cx. quinquefasciatus* genome scaffolds were eventually assembled to the chromosome level using Hi-C technology [8], the underlying sequence contigs remained short and their annotation required further improvement. Moreover, a comparison of chromosome-scale genome assemblies from the major evolutionary lineages of mosquitoes has never been performed.

Unlike *Drosophila* [30] and other insects [31], mosquitoes have a remarkably conserved karyotype, with the total number of chromosomes (2n) equaling 6 for nearly all mosquitoes. The only known exception is *Chagasia bathana* (Anophlenae subfamily), in which 2n equals 8 [32]. However, the sex chromosome/autosome arrangements differ between the two mosquito subfamilies. In the Anophelinae subfamily, the chromosomal complement consists of 2 heteromorphic sex chromosomes, X and Y, and 2 pairs of autosomes. Culicines, in contrast, have three pairs of autosomes, one of which contains a sex-determining locus. In the Anophelinae lineage, autosomes have undergone multiple autosomal arm translocations and the gene order within all chromosomes was significantly reshuffled due to multiple paracentric inversions [33]. In addition, a large inter-chromosome translocation was discovered between the mosquitoes from the Anophelinae and Culicinae subfamilies [28]. According to the current hypothesis, a part of chromosome 2, represented by the 2R arm in *An. gambiae*, was translocated to chromosome X resulting in the formation of homomorphic sex-determining autosome 1 that carries the male-sex determining locus M in *Cx. quinquefasciatus* [34] and *Ae. aegypti* [35]. Availability of chromosomal level assemblies for all major taxa of mosquitoes provide a unique opportunity to further investigate their chromosomal evolution.

Because only a female mosquito can bite and transmit diseases, studying the M-locus and other sex-determining genes may potentially provide the foundation for mosquito control methods that convert female mosquitoes into harmless males. In *Ae. aegypti*, a chromosome quotient approach was used to identify male-biased genes including the male-determining factor *Nix* [35, 36] and *myo-sex*, a gene that encodes a duplicated myosin heavy chain protein [37] important for male flight [36]. Both *Nix* and *myo-sex* are in a 1.3-Mbp-long M locus located near the centromere of chromosome 1, which also contains more than 20 non-coding RNA genes [21]. No such region has been reported for *Cx. quinquefasciatus*.

In this study, we used advanced genome technologies such as ONT sequencing, Hi-C scaffolding, Bionano optical mapping, and FISH-based cytogenetic mapping to develop a significantly improved *Cx. quinquefasciatus* genome assembly. This assembly was employed to reannotate several important gene families. A polymorphic inversion on chromosome 3 and a genomic region that contains male-specific sequences including a homolog of *myo-sex* on chromosome 1 were identified in the *Cx. quinquefasciatus* genome. We also apply this assembly together with three other available chromosome-scale assemblies for *An. coluzzii*, *An. albimanus*, and *Ae. aegypti* to better understand genome evolution in the major mosquito lineages and to clarify divergence times.

## Results

### Genome assembly development, metrics, and features

**Fig. 1:**
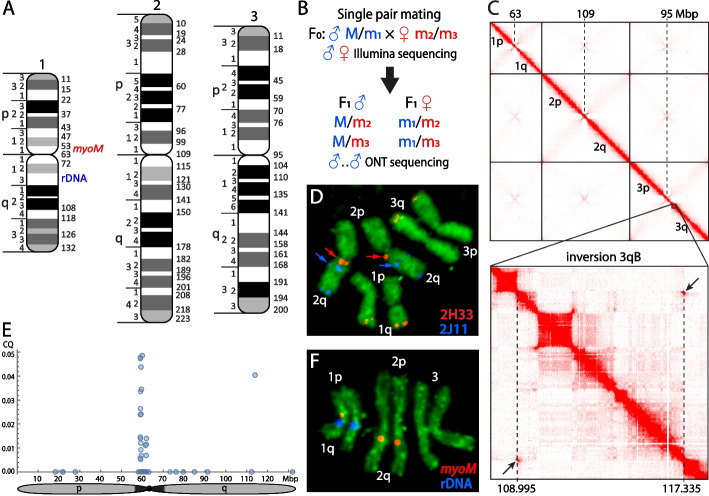
Physical mapping and Hi-C scaffolding of the *Culex quinquefasciatus* genome.** A** The physical genome map. Numbers 1, 2, and 3 above the chromosomes stand for the chromosome numbers and letters p and q indicate chromosomal arms. Cytogenetic regions of the chromosomes are shown at the left side of the idiograms. Genomic coordinates in Mb are shown at the right side of the idiograms.** B** The crossing scheme to generate data by TrioCanu. Only M/m loci are shown, m1–m3 refer to possible variants in m sequences. The two parents were sequenced separately using Illumina. F1 males were sequenced by ONT. **C** A final Hi-C heat map for the *Cx. quinquefasciatus* genome. The position of the chromosomal inversion 3Rb is indicated by a rectangle. “Butterfly” shaped structure associated with inversion 3Rb is indicated by arrows. Centromere and breakpoint positions are shown by dashed lines. **D** An example of fluorescence in situ hybridization on mitotic chromosomes. Arrows indicate positions of the two BAC clones of interest. Hybridization of three other BAC clones is seen as red signals on 1q and 3q arms and blue signals on 2q arm. **E** Chromosome quotient (CQ). Each dot indicates a 1-kb fragment that showed a CQ value less than 0.05, indicating male specificity. Fragments with CQ values higher than 0.05 are not shown. The analysis was performed using repeat masked sequences and details are described in the “ Methods” section. **F** Fluorescence in situ hybridization of *myoM* gene and rDNA in chromosomes of male. Red and blue signals of the probes indicate location of the probes for *myoM* gene, a homolog of the *Ae. aegypti* M locus gene *myo-sex*, and rDNA, respectively

We developed an improved genome assembly for the JHB strain of *Cx. quinquefasciatus* VPISU-Cqui_1.0_pri_paternal (J5) anchored to chromosomes [38] (Fig. 1A). The JHB strain, which was also used for the development of previous genome assemblies J2 [28] and J3 [8], maintains high levels of heterozygosity, posing a challenge for assembly construction. We turned this potential pitfall into an advantage by employing a trio-binning sequencing approach [39]*.* The concept of this approach is shown in Fig. 1B. A total of 139-Gb shotgun Illumina sequencing reads from the F0 parents and approximately 89-Gb-long ONT sequences from F1 male siblings were obtained (Fig. 1B). ONT sequences were separated into paternal and maternal reads based on Illumina sequences derived from the F0 father (45.8 Gb used) and mother (46.8 Gb used). All ONT paternal reads plus unseparated reads were used to obtain a paternal assembly by Canu [39], which was then polished using the F0 Illumina reads by Pilon [40].

The assembly was scaffolded using Bionano optical and physical maps [41], followed by Hi-C [20]. Hi-C analysis was performed based on 0.5 Gb raw data from the Hi-C libraries, from which 0.32 G (64.7%) were unique. The scaffolding process is shown in Additional file 1: Fig. S1. The 3D-DNA application was employed to generate a draft assembly followed by visual inspection and ~150 manual corrections [42] that allowed scaffolds to be assigned to six chromosomal arms (1p, 1q, 2p, 2q, 3p, 3q). The chromosomal arms were further validated, oriented, and assembled to chromosome scale (Fig. 1C) using physical mapping data (Fig. 1A, Additional file 2: Table S1). Physical genome mapping was performed independently based on previously sequenced BAC clones from JHB colony [43] which were assigned to the mitotic chromosomes of *Cx. quinquefasciatus* (Fig. 1D). A total of 143 markers were mapped to chromosomes (Additional file 2: Table S1). The final physical map included 53 genome coordinates assigned to the boundaries between chromosomal bands (Fig. 1A).

Interestingly, a visual inspection of chromatin interactions in the Hi-C contact map allowed us to identify a large polymorphic inversion 3qB on chromosome 3 with a total length of 8.4 Mb. The heatmap showed a clear “butterfly” pattern far from the main diagonal where genomic positions of connecting points mark inversion breakpoints (Fig. 1C). Approximate breakpoint coordinates were determined as 108,955,000–117,355,000 bp on chromosomal arm 3q of the J5 genome. The resolution of the Hi-C map used for breakpoint identification was ~5 kb, which means these breakpoints carry an uncertainty of ±5kb. The described inversion appears to be polymorphic in JHB since interactions near the breakpoints on the main diagonal were not completely disrupted despite the increased number of interactions seen at the “butterfly” center [44]. This inversion was previously identified in a cytogenetic study of *Cx. quinquefasciatus* and was called the 3Rb inversion [45].

Our study identified a region that was enriched in male-specific sequences using the chromosome quotient (CQ) approach [46]. We sequenced pooled females and males from the JHB strain and mapped the resulting reads to the J5 assembly. As shown in Fig. 1E, a small region from ~58.9 to 61.9 Mb on chromosome 1 was enriched for male-specific fragments as indicated by a CQ less than 0.05. Interestingly, this region contains *myoM*, a homolog of the *Ae. aegypti* M locus gene *myo-sex* which is required for male flight [36, 37]. This gene was mapped to chromosomes using FISH in the region 1p11 close to the centromere and also on arm 2q where an autosomal paralog of this gene was also found in the genome (Fig. 1F). The chromosome 1 signal was seen only on the male-determining copy of chromosome 1; it was missing on the female copy of chromosome 1. However, sequences homologous to the *Ae. aegypti* male-determining factor *Nix* [35, 36] were not found in this region nor any other location in the genome. Although the region between 58.9 and 61.9 Mb likely includes the M locus in *Cx. quinquefasciatus*, the precise borders of the M locus remain to be determined and will likely require comparison of haplotype-resolved paternal and maternal assemblies.

### Gene content of the new assembly

**Fig. 2:**
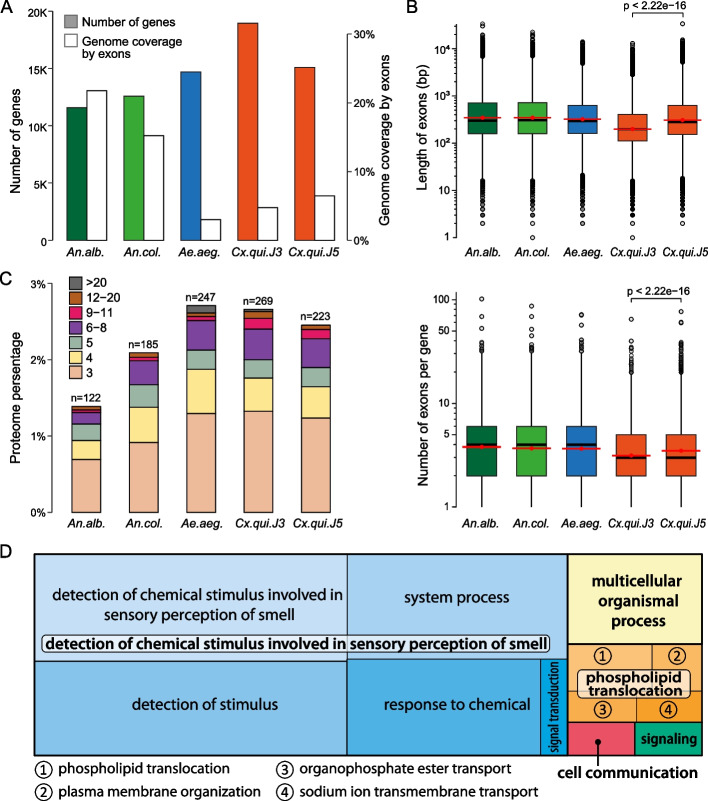
*Culex quinquefasciatus* J3 and J5 genome comparison and gene evolution in mosquitoes. **A** The abundance of protein-coding genes and the percentage of their exon coverage in the genome assemblies of four mosquito species. The abundance of genes and exon coverage are shown as green and orange bars, respectively. **B** The boxplots of the exon lengths of the protein-coding genes (top) and the numbers of exons per one protein-coding gene (bottom) in the log2-scale for the genomic assemblies of four mosquito species. Red dots indicate the mean values, and red error bars indicate standard errors of the mean values. The statistical significance of differences was measured with the unpaired Wilcoxon test. **C** The percentage of the proteomes in the genomes of four mosquito species including J3 and J5 assemblies of *Cx. quinquefasciatus*, occupied by members of the gene families (see “ Methods”). Proteins were included to the same family if their identity to each other were ≥50% along ≥70% of their lengths. Only families with at least three members are shown. **D** Gene ontology (GO) for protein families identified as expanded for *Cx. quinquefasciatus* in relation to other dipterans (see “ Methods”)

The new assembly was annotated by NCBI with the automated RefSeq Eukaryotic Genome Annotation Pipeline [47] using 2.8 billion reads from 46 RNA-seq runs and proteins from mosquitoes and other insects as evidence. Substantial improvements were noted for the J5 assembly over the previous J2 [28] and J3 [8] assemblies in terms of contiguity (N50: the length of the shortest read in the group of longest sequences that together represent at least 50% of the nucleotides in the set of sequences), completeness (BUSCO: Benchmarking Universal Single-Copy Orthologs), and accuracy (QV: Quality Value) scores (Table 1). The total of 15,081 protein-coding genes were identified in the new J5 genome assembly. While this represents a 20% decrease compared to the annotation of the prior J2 assembly [28], the new annotation is a considerable improvement by several metrics. Average protein length is increased by 26% (from 436 aa to 550 aa) when considering the longest protein per gene. For protein-coding genes, overall exon coverage of the genome (6.4 vs 4.7%, Fig. 2A), median exon length (284 bp vs 199 bp, Fig. 2B top), and average number of exons per gene (4.4 vs 3.8, Fig. 2B bottom) are all increased compared to the previous release. Alternate splice isoforms and untranslated regions (UTRs) were also added. The new annotation is more complete as measured by BUSCO (Table 1) and is comparable to two other mosquitos [21, 48] annotated by NCBI as shown in the assembly report based on BLASTp (Basic Local Alignment Search Tool, protein) alignments to *Drosophila* proteins [49]. Part of the decrease in protein-coding gene count is explained by incorporating 2935 old genes into others as part of the annotation process, reflecting a mixture of combining partial annotations (previously on different scaffolds or neighboring on the same scaffold) and redundant haplotypes. To further support that the fraction of gene models from the current genome resulted from the merging of gene models from the previous assembly, we compared the number of protein products that were composed of 3 or more protein products in the old and new versions of the genome annotation (Fig. 2C). We found that 824 gene models in the current J5 genome corresponded to 1888 gene models from J3 assembly while only 161 models of J5 derived from splitting of 72 genes from the previous J3 assembly. Thus, the merging of gene models can explain the absence of 983 of the ~3.8k genes originally present in the older assembly. In addition, we examined multi-gene families in the proteomes of J3 and J5 assemblies of *Cx. quinquefasciatus* in comparison to the proteomes of *Ae. aegypti*,* An. albimanus*, and *An. coluzzii*. We found that the proportion of the multi-gene families with at least three members is comparable between J3 (2.7%), J5 (2.5%), and *Ae. aegypti* (2.7%) proteomes, and exceed the same fraction in *An. albimanus* (1.4%) and *An. coluzzii* (2.1%) (Fig. 2C). Therefore, our analysis does not support the previously proposed idea that multigene families have expanded significantly in *Cx. quinquefasciatus* when compared to *Ae. aegypti* [28]. Finally, detailed analysis of the tRNA genes indicated that the new assembly provided additional sequences that were not present in the older assemblies (Additional file 1: Fig. S2A).

**Table 1:** Quality comparison of the Cx. quinquefasciatus genome assemblies. J2 represents the original assembly [28], which was then scaffolded using Hi-C data to generate J3 [8]. J5 is the new genome assembly presented here

Assembly	BUSCO v3.0.2 diptera_odb10	Contig N50	Scaffold N50	Total Length	QV
J2	C:93.9%[S:89.9%,D:4.0%],F:1.7%,M:4.4%,n:3285	28,546	486,756	579,042,118	19.495
J3 all scaffolds	C:93.5%[S:91.2%,D:2.3%],F:1.9%,M:4.6%,n:3285	28,546	190,989,159	560,547,476	18.921
J3 chromosomes	C:91.1%[S:90.2%,D:0.9%],F:1.7%,M:7.2%,n:3285	28,546	190,989,159	523,187,415	18.905
J5 all scaffolds	C:93.8%[S:93.1%,D:0.7%],F:0.8%,M:5.4%,n:3285	2,875,282	201,550,677	573,230,032	29.201
J5 chromosomes	C:93.6%[S:92.9%,D:0.7%],F:0.8%,M:5.6%,n:3285	2,875,282	201,550,677	559,588,684	29.201

With the improved *Cx. quinquefasciatus* genome annotation in hand, we conducted orthology-based analyses to identify a core set of genes that are present in *Cx. quinquefasciatus* but not in four other mosquito species nor in the vinegar fly *D. melanogaster*. These unique genes are enriched for those involved in chemical sensation (Fig. 2D), which is unsurprising given the high variability and rapid evolution of genes associated with chemical reception in insects [50], as well as several other biological processes.

### Genome quality validation

**Fig. 3:**
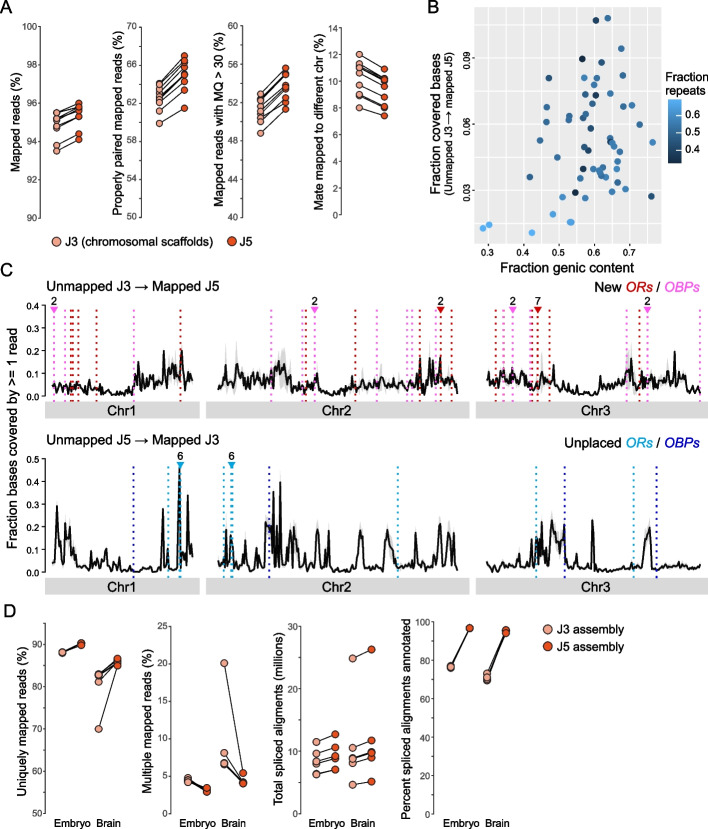
Improved mappability of RNA and gDNA sequencing data to the new assembly for *Cx. quinquefasciatus*. **A** Mapping of short genomic reads from field-caught *Cx. quinquefasciatus* mosquitoes to the chromosomal scaffolds of the new (J5) and old (J3) assemblies. Percentages of reads that mapped in various ways. Each set of paired dots with connecting line represents the reads from a single individual. **P < 0.01 (Paired Wilcoxon singed-rank tests). **B**–**C** Analysis of genomic regions harboring “assembly-specific” reads, which mapped to only J5 or only J3. Panel **B** shows the genic and repeat content of such regions in the J5 assembly. Each dot represents a non-overlapping 10 Mb, and the *y*-axis shows the fraction of bases within the given window covered by reads that did not map to J3. Panel **C** shows the distribution along chromosomes of J5-specific (top) or J3-specific (bottom) reads. J3 chromosomes are 5–10% shorter than their J5 counterparts but plotted proportionally to take up the same total space in the panel. Vertical, colored, dotted lines show the locations of odorant receptors (*ORs*) and odorant-binding proteins (*OBPs*) that were new or unplaced in J5. Note that only 17 of 19 unplaced *ORs* were located on the chromosomal scaffolds of J3 and are thus plotted at the bottom. Numbers above the lines indicate that the given number of new or unplaced genes is present at that location. **D** Mapping of RNAseq reads from embryo [51] and adult brain [52] to the new and old assemblies. Each set of paired dots with connecting line represents the reads from a single sample. Embryonic samples include 2 from the anterior pole and 3 from the posterior pole. Brain samples include 3 females and 3 males

To further evaluate the new assembly, we collected whole-genome shotgun sequencing reads from ten *Cx. quinquefasciatus* mosquitoes recently captured in southern California (Additional file 2: Table S2) and mapped them to the three chromosomal scaffolds of both the J3 and J5 assemblies. More reads mapped to the J5 assembly than to the J3 assembly when considering all reads, properly paired reads, or reads with high mapping quality (Fig. 3A). Moreover, fewer mate pairs mapped to different chromosomes in the J5 assembly (Fig. 3A). Analysis of reads that mapped to only the J5 assembly spanned regions with moderate to high genic content and low to moderate repeat content (Fig. 3B) and were spread relatively evenly across each chromosome (Fig. 3C top), likely reflecting the substantial improvement in contiguity (Table 1). Reads that mapped only to J3 tended to congregate in particular places along each chromosome (Fig. 3C bottom), suggesting the presence of several discrete gaps in the new assembly and thus room for further improvement.

To assess this new annotation, we directly compared the quality of RNA-seq alignments to J3 and J5 annotations using data from two previous studies [51, 52]. For both datasets, a higher proportion of reads uniquely mapped to annotated genes in the current assembly relative to the previous assembly (Fig. 3D, additional file 1: Fig. S3A) and more genes were identified as differentially expressed (Fig. 3D, Additional file 1: Fig. S3B, D). We also observed a two-fold decrease in the proportion of multiple-mapped reads (from 7.1 to 3.8% on average for all datasets), suggesting that the previous assembly contained alternative haplotypes or models which split among smaller contigs or scaffolds. Moreover, mapping of RNA-seq reads to the current genome yielded an increase in both the number of total spliced alignments and the number of spliced alignments that match annotated splice junctions (Fig. 3D, Additional file 1: Fig. S3A). Taken together, these analyses indicate that the current genome assembly and annotation provide a marked improvement over the previous genome release [8].

### Annotation of odorant receptor and odorant binding protein genes

**Fig. 4:**
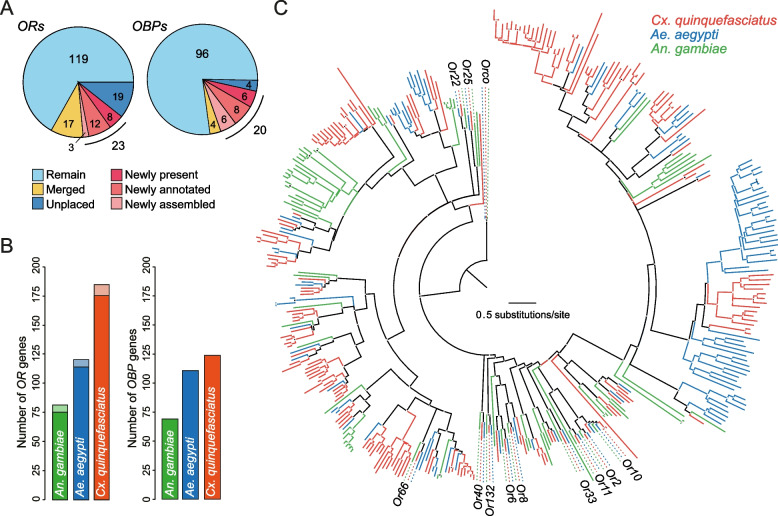
Odorant receptor and odorant binding protein genes in *Culex quinquefasciatus* and their evolution in mosquitoes.** A** Comparison of odorant receptors (*ORs*) and odorant-binding proteins (*OBPs*) annotated in current and previous assemblies. “Merged” genes represent cases where a single gene in the new assembly corresponds to 2–3 putative haplotypes present in the old assembly. “New” genes include *ORs* that were annotated on extrachromosomal contigs of J3 but have now been placed on a chromosome (newly assembled), *ORs* that were present in J3 but not annotated (newly annotated), and *ORs* that were completely absent from J3 (newly present). Unplaced genes were present in J3 but are absent from the new assembly. **B** Number of *ORs* and *OBPs* present in the genomes of three mosquito species. Dark and light sections of each bar correspond to full-length genes and fragments, respectively. **C** Inferred evolutionary relationships among *ORs* from three mosquito species. Maximum likelihood tree inferred using PhyML v3.0.0 based on translated protein sequences. The names of *ORs* with a single conserved ortholog in each species are labeled. See Additional file 1: Fig. S5 for tree with all gene names. Numbers of *OBPs* and *ORs* in *An. gambiae* and *Ae. aegypti* taken from [21, 33, 59]

We further assessed the new assembly via detailed reannotation of two large chemosensory gene families—odorant receptors (*ORs*) and odorant-binding proteins (*OBPs*)—with the help of newly collected bulk antennal and proboscis transcriptome data. These genes comprise some of the largest multigene families in insect genomes [53] and comparison of *OR* and *OBP* content in old and new assemblies can help highlight assembly improvements that likely also affect other genes. We identified 149 full-length, ligand-specific *OR* genes on the three chromosomal contigs of the new assembly, as well as 10 fragments encoding proteins <350 amino acids long (Additional file 1: Fig. S4). Two full-length genes (*Or105* and *Or205*) showed evidence of alternative splicing with distinct initial exons spliced to shared final exons—a pattern also seen in *ORs* from other insects [54]. When comparing the new annotations to the union of two previous *OR* annotation efforts [55, 56], we found that 119 of our ligand-specific *ORs* were also annotated as single genes on the chromosomal contigs of the J3 assembly (Fig. 4A, “remain”), while 17 correspond to two or three very similar genes in the previous annotation that likely represent alternative haplotypes (Fig. 4A, “merged”). We consider the remaining 23 *ORs* in our annotation set to be new because they were not present and/or annotated on the chromosomal scaffolds of the J3 assembly (Fig. 4A). Conversely, 19 *ORs* from the previous annotation were absent from the new assembly (Fig. 4A, “unplaced”). A search for these “unplaced” genes in the sequence data from earlier stages of the new assembly pipeline revealed that they exist in the raw data but were excluded by the program Canu during assembly of raw Oxford Nanopore reads into contigs. These “unplaced” genes were found in a draft assembly [57] obtained using the FLYE algorithm [58] and the paternal-specific ONT reads obtained from TrioCanu. The “unplaced” genes were excluded by the program Canu during assembly of raw Oxford Nanopore reads into contigs, not during subsequent scaffolding steps. Consistent with the idea that at least some of these genes are located in gaps in the new assembly, 14 of 19 unplaced *ORs* fall in narrow windows of the J3 assembly where gDNA reads from wild-caught mosquitoes mapped to the chromosomal scaffolds of J3, but not J5 (Fig. 3C bottom).

Taken together, the final tally of full-length, ligand-specific *ORs* in *Cx. quinquefasciatus* is 168 (149 genes present in J5 plus 19 unplaced genes). This represents a substantial gene family expansion relative to *Ae. aegypti* (*n*=113 full-length *ORs*), and *An. gambiae* (*n*=75 full-length *ORs*) (Fig. 4B), corroborating the analysis of novel gene content (Fig. 3D). We inferred the phylogenetic relationships among *Cx. quinquefasciatus ORs* alongside those from the other two mosquito species, revealing many *Culex*-specific expansions, in addition to 11 *ORs* with 1-to-1 orthologs in all three species (Fig. 4C, Additional file 1: Fig. S5). To facilitate future functional studies, the *Cx. quinquefasciatus* names for these 11 highly conserved *ORs* were changed to match those used in the other mosquitoes (see Additional file 2: Table S3 for renaming details). Metadata, coding sequences, proteins sequences, and annotation details for all *ORs* can be found in the Additional files (Additional file 2: Table S3, metadata; Additional file 3, coding sequences; Additional file 4, protein sequences, Additional file 5, annotation gff).

Annotation of *OBPs* in the J5 assembly also revealed genome improvements, through identification of novel genes and merging of allelic variants (Fig. 4A). One hundred and sixteen *OBPs* were identified on the three *Cx. quinquefasciatus* chromosomes (Fig. 4A,B, Additional file 1: Fig. 4). Of these, 101 *OBPs* present in J3 chromosomes or extra-chromosomal scaffolds, were also present in the new assembly. Twelve were newly identified and could be classified as classic (*n* = 4), plusC (*n* = 3), minusC (*n* = 2), and atypical (*n* = 3) according to the conserved number and spacing of cysteine residues in the peptide sequence [59, 60] (Additional file 2: Table S4). Three of the previously described *OBPs* (18, 30, and 56) each aligned to two distinct regions of the new assembly with high sequence similarity. The two copies identified for *OBPs* 18 and 56 were found in tandem, consistent with a recent duplication event, yet the copies of *OBP30* appeared on separate chromosomes. Four pairs of previously described *OBPs* were identified as alternate alleles found on redundant haplotypes in the old assembly (*OBPs* 38-40, 54), and were merged with *OBPs* 33-36 in the new assembly. Altogether, this resulted in a total of 101 *OBPs* present as a single gene in J3, 15 newly annotated (12 newly identified + 3 duplicate) *OBPs*, and 4 merged *OBPs* (Fig. 4A, right). Four of the previously described 109 *OBPs* could not be located in the new assembly. As in the case of the missing *OR* genes, these *OBP* genes were found in the raw reads but excluded during Canu assembly. Assembly improvements and RNA-sequencing data also allowed for identification of new *OBP* transcript variants. Two novel *OBPs*, 118 and 121 each had 2 transcript variants, and 7 of the original 109 *OBPs* had transcript variants that were predicted by RefSeq annotation. Former *OBP 70* had high sequence similarity to *OBP 69* in the new assembly and was designated a transcript variant (OBP 69_v2). As a resource for the community, we used the new *OR* and *OBP* annotations to estimate gene expression in chemosensory tissues from male and female mosquitoes using new and previously published RNA-seq data (Additional file 1: Fig. S6, S7, Additional file 2: Table S5).

### Genome content evolution in mosquitoes

**Fig. 5:**
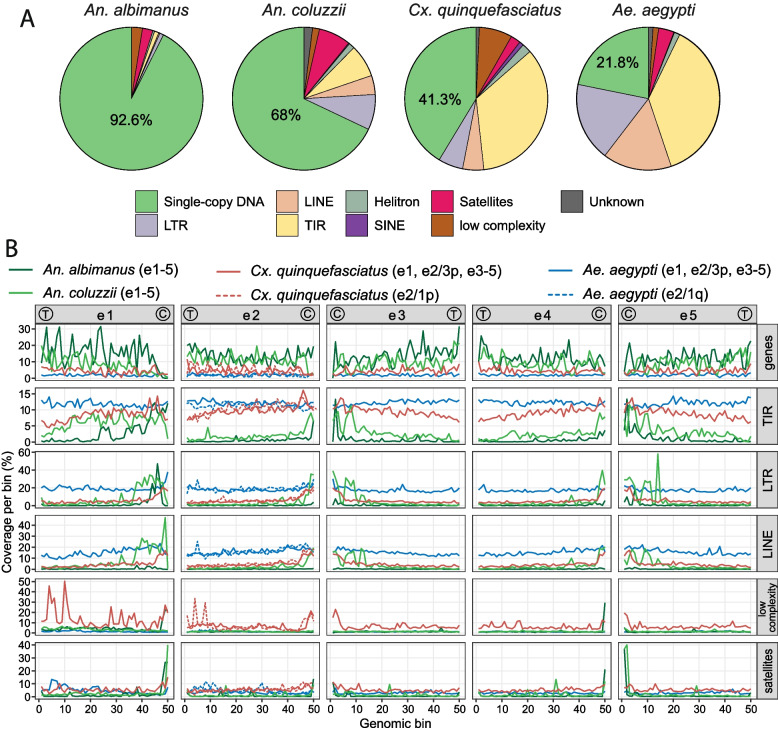
Genome evolution in mosquitoes. **A** Genome contents in mosquitoes. The pie diagrams illustrate the proportions of the single-copy and repetitive elements in the assemblies of the genomes for *An. albimanus*, *An. coluzzii*, *Cx*. *quinquefasciatus*, and *Ae. aegypti.* **B** Genome landscapes in mosquitoes. Profiles of the genomic content of genes, transposable elements, low-complexity regions, and satellites, along the chromosomal elements of four mosquito species are shown by lines with different colors. Each chromosomal element is split into 50 bins of different lengths, and the coverage of genetic elements occupying each bin is shown along the chromosomes. Positions of centromeres and telomeres are specified by letters C and T, respectively. Correspondence between chromosomal elements and chromosomal arms in mosquito species is shown in Fig. 6. Genome landscape in 1p and 1q arms of *Cx. quinquefasciatus* and *Ae. aegypti* which correspond to the element e2 in *An. coluzzii* are shown by dashed lines

We used the new assembly to compare the abundance and chromosomal distribution of various genomic features, such as genes, TEs, low-complexity DNA, and satellites in *Cx. quinquefasciatus* (Additional file 1: Fig. S8), *An. coluzzi* [18], *An. albimanus* [17], and *Ae. aegypti* [21] (Fig. 5A). The proportion of single-copy DNA, which does not harbor repeats, was much higher in the *An. albimanus* (92.6%) and *An. coluzzii* (68%) genomes than in *Cx. quinquefasciatus* (41.3%) and *Ae. aegypti* (21.8%). To analyze the distribution of various genomic features along the chromosomes, the chromosomal arms of all four mosquitoes were divided into a set of equal number of bins. We used traditional chromosomal element nomenclature, wherein the chromosomal arms of *An. coluzzi* X, 2R, 2L, 3R, and 3L were considered as chromosomal elements e1, e2, e3, e4, and e5, respectively [33]. Genomic features were compared across bins on the homologous chromosomal elements. Among all features, only genes were distributed relatively evenly along chromosomes with a slight decrease in gene density in pericentromeric regions for all species (Fig. 5B).

To explore the basis of the large differences in genome size among mosquitoes, we identified and annotated TEs, satellites, and low-complexity elements in the new *Cx. quinquefasciatus* assembly (Additional file 1: Fig. S8) and the three other species (Fig. 5). The study included both the known TE families from RepBase [61] and TEfam [62] databases and novel TE families discovered using the EDTA pipeline [63] for all species. In agreement with previous observations [21, 28, 64], we found that the repeat fraction of the genome varied drastically across the mosquitoes, reaching 7.4, 32, 58.7, and 78.7%, in *An. albimanus*, *An. coluzzi*, *Cx. quinquefasciatus*, and *Ae. aegypti*, respectively (Fig. 5A). Although the elevation of the TE content occurred among the genomes in the Anophelinae subfamily, the most significant TE expansion took place in the Culicines. DNA transposons with terminal inverted repeat sequences (TIRs), including MITEs (Miniature Inverted-repeat Transposable Elements), have undergone the most dramatic increase in the genomes of both culicines *Cx. quinquefasciatus* (35% of the genome) and *Ae. aegypti* (38% of the genome). For comparison, TIRs, including MITEs occupied only 1 and 7.4% of the *An. albimanus* and *An. coluzzi* genomes, respectively. Among the most abundant TIR’s superfamilies were Sola (6.5%) and hAT (5%) in *Cx. quinquefasciatus* and MULE-MuDR (5.9%) and hAT (4.9%) in *Ae. aegypti* (Additional file 1: Fig. S9). In contrast, the LTR (Long Terminal Repeats) and LINE (Long Interspersed Nuclear Elements) retrotransposons had a relatively low density in the *Cx. quinquefasciatus* genome (4% of LTRs and 4.7% of LINEs) than in *Ae. aegypti* (15.8 and 15.5%, Fig. 5). The same was true for Copia elements that covered only 0.3% of the *Cx. quinquefasciatus* genome but 0.8 and 5.1% of *An. coluzzi* and *Ae. aegypti* genomes (Additional file 1: Fig. S9). The genome of *Cx. quinquefasciatus* contained up to 7.5% low-complexity regions, characterized by stretches of one or two out of four possible nucleotides, while the other genomes contained no more than 2.5%. Satellites, composed of extended arrays of tandemly repeating non-coding DNA, were slightly enriched only in *An. coluzzi*, covering 7.3% of its genome in comparison to 2.2–3.5% of the other mosquito genomes.

Similar to the gene distribution, we examined the distribution of repeats along chromosomes. Unlike genes, the distribution of repeats differed remarkably among the species. In the genomes of both *Anophelines*, TIR transposons were located mostly in pericentromeric areas with 5–10% coverage per bin, while reaching only 3% coverage per bin in euchromatic regions (Fig. 5B). In *Cx. quinquefasciatus*, TIRs were distributed across the whole chromosomes, but their density gradually decreases from pericentromeric regions (10–15% per bin) toward telomeres (7% per bin). In contrast, TIRs were evenly distributed along the chromosomes of *Ae. aegypti* at the level of ~13% per bin without any enrichment near the pericentromeric regions. The distribution of LTR and LINE retrotransposons across mosquito chromosomes was similar to TIRs (Fig. 5B). They were highly enriched in the pericentromeric regions of *Anopheles*, while their accumulation is less pronounced in the pericentromeric regions of *Cx. quinquefasciatus*. LTR and LINE elements were evenly distributed along the chromosomes at the same levels of ~15% per bin without enrichment at the pericentromeric regions of *Ae. aegypti*. It is worth mentioning that the pericentromeric regions of *Cx. quinquefasciatus* also have a high abundance of low-complexity repeats. In contrast, pericentromeric regions in *Anopheles* species were enriched in satellites (Fig. 5B).

We also investigated whether the TE expansion in the Culicinae genomes occurred by transposon family diversification or by proliferation of existing families (Additional file 1: Fig. S9). Some DNA transposons, such as CMC, hAT, MuDR-MULE, and some LTR retrotransposon, such as Bel-Pao, evolved by increase in both the genome coverage and a number of families from *An. albminanus* to *An. coluzzii*, then to *Cx. quinquefasciatus*, and finally to *Ae. aegypti*. For example, the CMC superfamily was represented by 19, 70, 134, and 353 families that covered 0.13, 0.56, 2, and 4% in the genomes of *An. albminanus*, *An. coluzzii*, *Cx. quinquefasciatus*, and *Ae. aegypti*, respectively. In contrast, the DNA transposon Sola had lower diversity in the genome of *Cx. quinquefasciatus* (8 families) relative to that of *Ae. aegypti* (14 families) but as a result of proliferation this element covered 6.5% in the *Cx. quinquefasciatus* genome but only 2.9% of the *Ae. aegypti* genome. The 5-fold increase in the genome coverage of LINE retrotransposons RTE and R1 between *An. coluzzii* and *Ae. aegypti* from 0.8 and 0.75% to 3.1 and 4%, respectively, was accompanied by only a minor difference in family diversity. Thus, both the appearance of new transposon families and the proliferation of existing families were involved in increasing TE abundance in culicine genomes (Additional file 1: Fig. S9).

Interestingly, a comparison of tRNAs between the mosquitoes and other Dipteran species indicated much higher numbers of predicted tRNA genes in species of *Culex* and *Aedes* versus *Anopheles* and other species of Diptera (Additional file 1: Fig. S2B). tRNAs linked to specific isotypes showed a similar trend as the total predicted tRNA genes were mostly linked to a specific amino acid, except for selenocysteine that was enriched in *Culex* and *Aedes* (Additional file 1: Fig. S2C). Lastly, at the anticodon levels, a general enrichment occurred in *Culex* and *Aedes* compared to other mosquitoes and fly species outside of *Stomoxys calcitrans* (Additional file 1: Fig. S2D), but it was anticodon specific that likely reflects codon bias for each species.

### Phylogeny and chromosomal evolution in mosquitoes

**Fig. 6:**
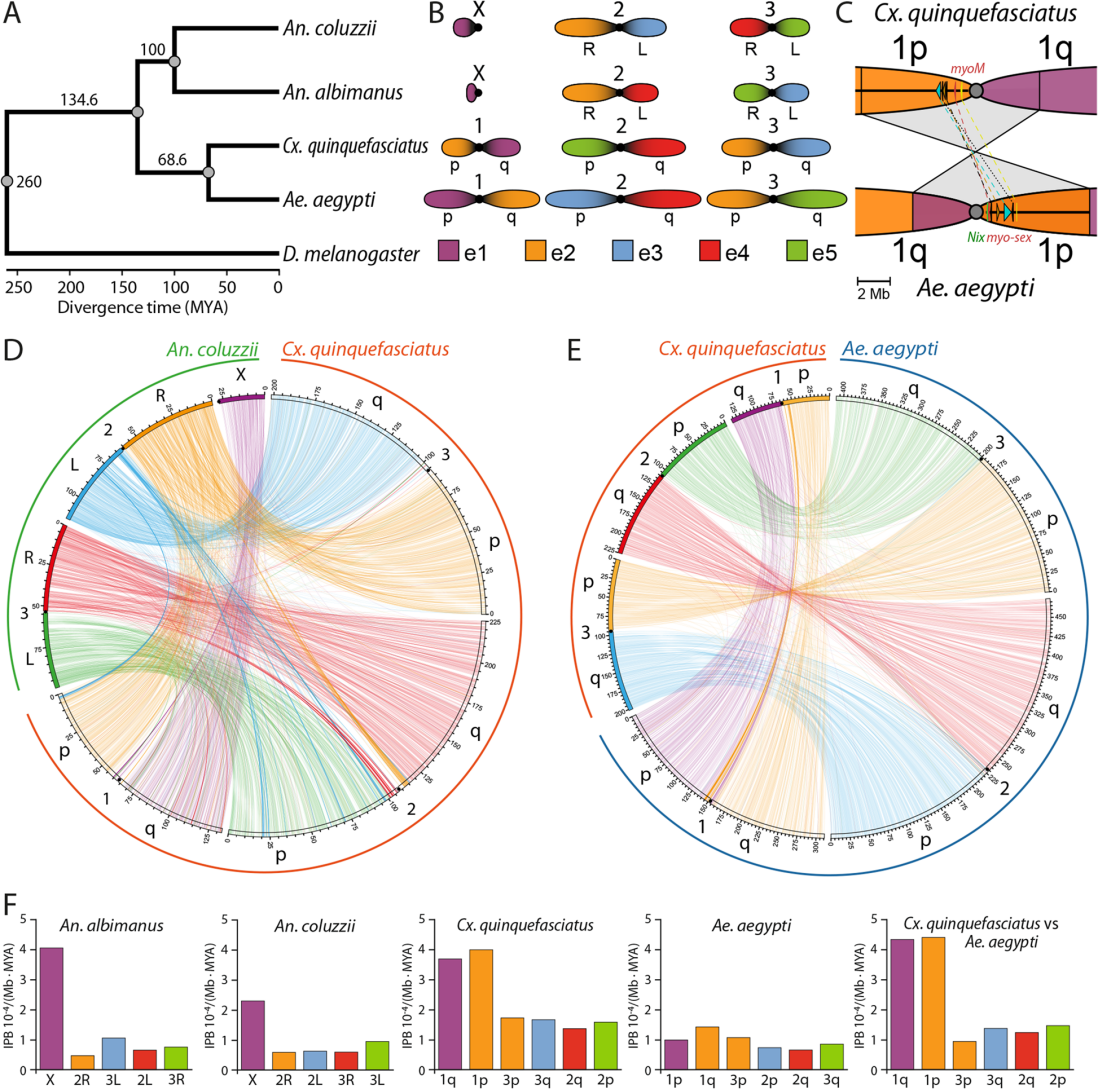
Chromosomal evolution in mosquitoes.** A** The estimated maximum likelihood molecular phylogeny of mosquitos. *D. melanogaster* was used as the outgroup species. The scale indices the divergence time in million years. **B** The reshuffling of the chromosome elements in the mosquito karyotypes. Chromosomal elements are indicated by different colors according to previously published nomenclature for the *Anopheles* species [33]. Chromosomes and chromosomal arms are shown by number 1, 2, 3, and letters p (short) and q (long), respectively. The lengths of the element are shown in proportions of the real chromosome length measurements [29, 66, 67]. **C** The pericentric inversion in the chromosome 1 between *Cx. quinquefasciatus* and *Ae. aegypti. Nix*, and *myo-sex* are genes described in the M locus of *Ae. aegypti* [21]. *Myo-M* is a new gene identified in *Cx. quinquefasciatus* genome. **D** Chromosomal syntheny plots between *An. coluzii* and *Cx. quinquefasciatus* species based on the single-copy orthologs. Each line represents a single gene ortholog. Lines are colored according to the chromosome elements. **E** Chromosomal syntheny plots between *Ae. aegypti* and *Cx. quinquefasciatus* species based on the single-copy orthologs.** F** The average rate of the rearrangements between the chromosome elements. First three charts show the rates of rearrangements between different mosquito species and the common ancestor. The last chart shows the rate of rearrangements between *Cx. quinquefasciatus* and *Ae. aegypti*. Rates are measured as breaks between syntenic blocks per Mb per million years (MYA)

We took advantage of the chromosome-level genome assemblies of *Cx. quinquefasciatus*, *An. coluzzi* [18], *An. albimanus* [17], and *Ae. aegypti* [21] to rebuild the phylogeny and estimate more precisely the evolutionary distances among mosquitoes from two major mosquito subfamilies Anophelinae and Culicainae, and two tribes Culicini and Aedini. We applied maximum likelihood phylogenetic analysis to multiple alignments of 2842 universal single-copy protein orthologs revealed by BUSCO. The time calibrated tree demonstrated that Culicinae and Anophelinae diverged ~136 MYA while *Cx. quinquefasciatus* and *Ae. aegypti* had a common ancestor ~69 MYA (Fig. 6A). In comparison, another estimation based on phylogenomic analysis of 709 single-copy ortholog groups from 256 mosquito species assessed the divergence time between Culicinae and Anophelinae subfamilies as ~147–213 MYA and Culicini and Aedeomyiini tribes as ~109–162 MYA [65].

The genome analyses provide some new insights into chromosomal rearrangements in *Cx. quinquefasciatus* and other mosquitoes. We compared the details of chromosomal evolution among the species from divergent evolutionary lineages: *Cx. quinquefasciatus*, *Ae. aegypti*, *An. coluzzi*, and *An. albimanus* [33]. Single-copy orthologs were then identified and the genomic positions and orientation of conserved syntenic blocks were determined. Here, we defined a conserved syntenic block as a locus in which at least two single-copy orthologs identified by BUSCO analysis are located near each other with conserved relative orientation. In total, we found 2842 shared orthologs forming 1415 non-overlapping anchor points that were arranged into 504 conserved syntenic blocks. The correspondence between the chromosomal elements in *Cx. quinquefasciatus* and the other mosquitoes included in this study were determined based on reshuffling of the conserved syntenic blocks.

A scheme of chromosomal rearrangements between the species is shown in Fig. 6B. In this figure, the chromosomal arms X, 2R, 2L, 3R, and 3L of *An. coluzzi* are traditionally considered as chromosomal elements e1, e2, e3, e4, and e5, respectively, and labeled with different colors [33]. Our study supports the previous observation that whole-arm translocations are common across different mosquito lineages [33]. For example, autosomal arm exchanges between *An. albimanus* and *An. coluzzii* [15] and between *Cx. quinquefasciatus* and *Ae. aegypti* [27] were confirmed. Accordingly, *An. coluzzii* and *Cx. quinquefasciatus* have the e2+e3 and e4+e5 autosomal arm associations, *An. albimanus* has e2+e4 and e3+e5 associations, while *Ae. aegypti* has e3+e4 and e2+e5 associations. In addition, *Cx. quinquefasciatus* and *Ae. aegypti* have e1+e2 association, which is not present in *Anopheles*. The comparison between Culicinae and Anophelinae revealed that chromosome arm 2R (e2) in *An. coluzzi* is a mosaic of 1p and 3p fragments from *Cx. quinquefasciatus*, while the other chromosomal arms are mostly homologous among the species. Analysis of species pairs: *Ae. aegypti* versus *An. coluzzii*, *An. albimanus* versus *An. coluzzii*, and *An. albimanus* versus *An. coluzzi* (Fig. 6C, D, Additional file 1: Fig. S10A) demonstrated that the mosaicism of e2 occurred before the split of *An. coluzzi* and *An. albimanus* suggesting either a whole-arm translocation from the chromosome 1 in Culicinae genome to another chromosome (current 2R arm or e2 of *An. gambiae*) or a translocation of a part of the current 2R arm (e2) in *An. gambiae* to the X chromosome (e1) forming two-arm chromosome 1 in Culicinae.

In this study, we discovered a new rearrangement in chromosome 1, a pericentric inversion between the arms of *Cx. quinquefasciatus* and *Ae. aegypti* (Fig. 6C). The size of this inversion equals 9.3 Mb and 11.2 Mb in *Cx. quinquefasciatus* and *Ae. aegypti*, respectively. This chromosomal rearrangement changed the position of the *MyoM* gene in *Cx. quinquefasciatus* compared to a homologous *Myo-sex* gene in the male sex-determining locus M in *Ae. aegypti* [21]. As a result, this gene was moved from 1p arm (e1) in *Ae. aegypti* to the nonhomologous 1p (e2) arm in *Cx. quinquefasciatus* (Fig. 6C)*.* Although the ribosomal locus is not resolved in the current *Cx. quinquefasciatus* assembly, the same rearrangement likely changed the position of the ribosomal locus that localizes in nonhomologous arms 1q in both species [29, 64].

In addition to inter-chromosome and inter-arm rearrangements, our study identified multiple paracentric inversions that reshuffled gene order within chromosomal arms of the studied species, the number of which varied across lineages (Additional file 1: Fig. S10). Here, we determined the rates of rearrangement for each of the four mosquito lineages. First, we reconstructed the order of the syntenic blocks in each arm in the common ancestor of *An. coluzzi*, *An. albimanus*, *Cx. quinquefasciatus*, and *Ae. aegypti* [68]. Second, we calculated the rate of the rearrangements for each arm as the ratio of inversion frequency along each lineage and the estimated divergence time from the common ancestor equal to 136 MYA (Fig. 6F). We estimate that the X chromosome has evolved 5.6 and 3.4 times more rapidly than autosomes in the *An. albimanus* and *An. coluzzi* lineages, respectively (Fig. 6F) supporting previous work [16, 33, 69, 70]. The sex-determining chromosome 1 has also evolved at a higher rate than other chromosomes in the Culicines—particularly in *Cx. quinquefasciatus*. Chromosome arms 1p and 1q evolved 2.5 and 2.3 times more rapidly than the other arms in *Cx. quinquefasciatus*, but only 1.2 and 1.7 times more rapidly than the other chromosomes in *Ae. aegypti* (Fig. 6F). This smaller difference in the rate of evolution between the chromosome 1 and autosomes in *Culicinae* species was also demonstrated by the paired comparison of rearrangement rates between *Cx. quinquefasciatus* and *Ae. aegypti* taking into account their estimated divergence time equal to 62.3 MYA (Fig. 6F, right panel). The paired comparison also revealed that arm 3p, a part of e2 after inter-chromosomal rearrangement during divergence between Anophelinae and Culicinae, has the lowest number of inversions relative to other arms (Additional file 1: Fig. S10B). In fact, this is the only chromosome arm that has three extended regions of syntenic blocks in the same order in both *Cx. quinquefasciatus* and *Ae. aegypti* (Additional file 1: Fig. S10B). These regions encompass ~50% of the chromosome arm and indicate that 3p has experienced fewer rearrangements than other chromosome arms after the split between the two species.

## Discussion

High-quality chromosome-scale genome assemblies are fundamental for the development of novel genetics-based vector control strategies [21]. The mosquito *Cx. quinquefasciatus*, the major vector of West Nile virus, has a genome with high levels of heterozygosity, enriched with repetitive DNA sequences, and lacking high-quality polytene chromosomes [28]. All these features make sequencing, assembling, and mapping of the genome challenging. In this study, the combination of Illumina and ONT sequencing, Hi-C scaffolding, Bionano, and cytogenetic mapping allowed us to overcome limitations of individual techniques and create an improved, chromosome-scale genome assembly for *Cx. quinquefasciatus* (Fig. 1). Specifically, the combination of ONT long reads and Illumina short reads allowed us to generate long contigs that were then polished with more accurate short reads. The use of trio binning [39] turned the high heterozygosity problem into an advantage in assembling the paternal genome. As shown in Table 1, the improvement was demonstrated in the completeness, contiguity, and accuracy of the current assembly. The integration of Bionano, Hi-C, and cytogenetics allowed verification of the scaffolding and further increased the accuracy of the assembly with 97.67% genome placement to chromosome positions. However, some gaps likely remain in the current J5 genome assembly, as revealed by the identification of small number of chemosensory genes that were annotated in the previous assembly but cannot be found in the current version of the genome.

The combination of long-read sequencing, Hi-C scaffolding, and physical mapping has proven to be successful for assembly of other mosquito genomes with high repeat content. For example, the AaegL5 genome assembly for *Ae. aegypti* was obtained by employing PacBio sequencing, Hi-C scaffolding, and physical mapping, resulting in placement of 94% of the genome along chromosomes [21]. Several chromosome-level *Anopheles* genomes were also successfully developed by combining short- and long-read sequencing data with Hi-C [17, 18, 20, 48], demonstrating the practical value of using Hi-C method as a reliable scaffolding tool. Overall, among all genome assemblies recently created for other mosquito species, the current J5 genome assembly of *Cx. quinquefasciatus* is the third after *An. albimanus* and *An. atroparvus* in terms of the percentage of the genome (97.67%) anchored to the chromosomes. For comparison, the percentage of the genome mapped to chromosome is 99.57 and 98.49% for the *An. albimanus, An. atroparvus,* [20]; 94% *Ae. aegypti* [21], 94.78% and 97.02% for *Cx. p. molestus* and *Cx. p. pallens* genomes [22]. These results highlight the importance of cytogenetic mapping for quality validation that have been performed specifically for these mosquito species.

Because the complexity of the *Culex* genome affects the quality of its polytene chromosomes, the identification of chromosomal rearrangements in this genus is difficult. Few inversions have been described in *Culex* [45, 71, 72]. In this study, Hi-C scaffolding led to identification of a polymorphic chromosomal inversion 3qB in the long arm of this chromosome in *Cx. quinquefasciatus*. We surmised that this inversion is polymorphic in the JHB strain, used for genome assembly, given the presence of an undisrupted main diagonal of contacts near the breakpoint loci in our Hi-C data (Fig. 1C). Thus, further investigation of this inversion in natural populations would be interesting. Previously, the development of a cytogenetic map for the salivary gland polytene chromosomes of the JHB strain of *Cx. quinquefasciatus* demonstrated the presence of two distinct paracentric chromosomal inversions [45]. One of them was 3Rb in subdivisions 58C–59C. Physical mapping based on FISH identified a marker CPIJ000458, which was found inside the inverted region. In the new J5 assembly, this gene is located within the inversion we identify with Hi-C on 3q. We therefore conclude that the inversion found in our Hi-C data is the same 3Rb inversion discovered by cytogenetic analysis. Although Hi-C is mostly used as an approach to characterize chromatin structure, we think that it also represents a reliable method for detecting chromosomal rearrangements in the genomes of Culicinae mosquitoes. In fact, this approach has already been used to detect and characterize chromosomal rearrangements in species of *Anopheles* [20, 44] and other organisms [73]. Thus, our study highlights the utility of the Hi-C approach for the identification of chromosomal inversions in the repeat-rich Culicinae genomes, which lack high-quality polytene chromosomes. Further application of this method will help us understand whether any chromosomal rearrangements are associated with species, subspecies, and/or ecotypes within the *Culex pipiens* complex [74] and thus potentially involved in adaptation of natural populations of these mosquitoes.

Our study identified a small region with the genome coordinates from ~58.9 to 61.9 Mb on chromosome 1 that is enriched for male-specific fragments (Fig. 1E) and contains *myoM*, a homolog of the *Ae. aegypti* M locus gene *myo-sex* [36, 37]. However, the precise borders of the M locus remain to be determined for *Cx. quinquefasciatus*. A homolog of the *Ae. aegypti* male-determining factor *Nix* was not found in this region or anywhere in the genome. In a recent survey of 14 species from 4 diverse tribes in the subfamily Culicinae, *Nix* has been found in all species except for *Cx. quinquefasciatus* [75]. The absence of *Nix* in *Cx. quinquefasciatus* was supported by analysis of raw reads, contigs, as well as assemblies [75]. The identity of the male-determining factor in *Cx. quinquefasciatus*, a basal member of the subfamily Culicinae, is a fascinating question that remains to be resolved.

The availability of a high-quality genome for *Cx. quinquefasciatus* in addition to other major mosquito taxa allowed us to uncover some interesting features of genome evolution in mosquitoes and provide insights into future perspectives in this field of studies. Our study confirmed a previously detected expansion of *ORs* and *OBPs* compared to other mosquito species, which could be related to the unique ecological or behavioral challenges faced by *Culex* mosquitoes or to changes in genome size and duplication dynamics (Fig. 4). Because the *Cx. quinquefasciatus* genome is about two times larger than that of *An. gambiae* [26] but less than half of the *Ae. aegypti* genome size [21], unique aspects of transposable element content, distribution, or recency in activity may have led to higher rates of *OR* duplication or lower rates of *OR* deletion. More functional studies will be necessary to understand exactly what fraction of the ~180 *ORs* found in *Cx. quinquefasciatus* are functionally expressed and relevant to behavior.

**Fig. 7:**
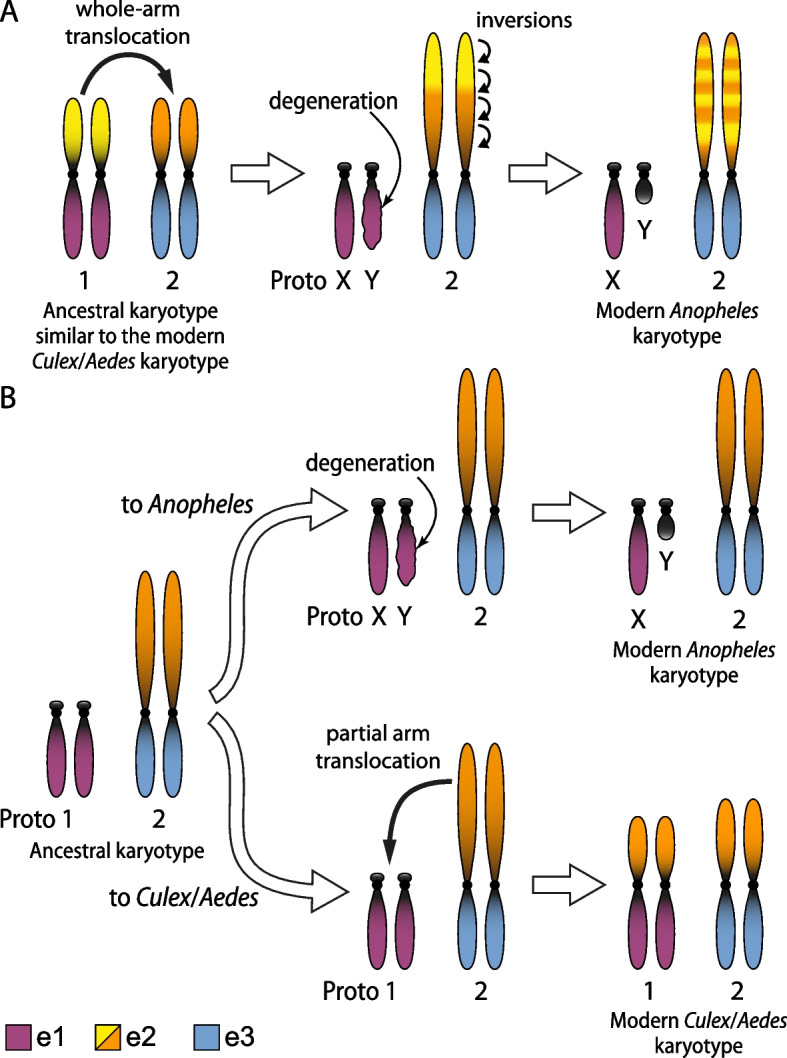
Chromosomal evolution in mosquitoes. **A** The scenario of whole-sex-chromosome arm translocation to the autosome in the Anophelinae lineage. **B** The scenario of partial autosomal arm translocation to the sex-determining chromosome in the Culicine lineage

Indeed, the most dramatic differences in genome evolution across four key taxa were related to the expansion of TEs in the genomes of Culicine mosquitoes (Fig. 5). Thus, we specifically focused on annotation and comparison of TEs for *An. albimanus*, *An. colluzzii*, *Cx. quinquefasciatus*, and *Ae. aegypti.* Previous analysis of earlier genomic assemblies for *An. albimanus*, *An. coluzzii*, and *Cx. quinquefasciatus* suggested that TEs comprise 2, 17.8, and 43.6% of the genomes, respectively [28, 33, 76]. Our analysis of improved assemblies has demonstrated similar or slightly higher TE content, at 2.3, 23.2, and 48.9%, respectively (Fig. 7). In contrast, the TE content in *Ae. aegypti* was significantly higher (73.4%) in our study than in the previously published report (54.9%) [21]. We think that this difference is related to our approach that predicted significantly more TIRs (37% vs 21%) and LTR (17.8% vs 12%) transposons. The previous annotation of the novel TEs in the *Ae. aegypti* genome was performed with RepeatModeler (v.1.08) software that utilized a homology-based approach for TE discovery [21] and was unable to effectively predict LTR and TIR transposons [63, 77]. Instead, we used the EDTA pipeline that includes several structurally based modules that allow it to discover TIRs and LTR transposons with high sensitivity [63].

The expansion of transposons in a genome is widely considered to be harmful to an organism and therefore expected to be prevented by innate defense systems [78]. This raises the question of why Culicinae mosquitoes are tolerant to the increase of transposons in their genomes? Extensive studies conducted mostly on *D. melanogaster* have elucidated the powerful system preventing transposon and virus propagation. The core activity of this system includes small non-coding RNAs, mainly Piwi-interacting RNAs (piRNAs), and proteins from the Argonaute (Ago) family. piRNAs guide PIWI proteins from the Ago family to cleave target RNA, methylate DNA, and promote heterochromatin assembly [79, 80]. piRNAs were originally identified as factors that have the ability to silence transposons but later their function as suppressors of endogenous protein-coding genes and virus expression was also discovered [81–83]. There is another class of small RNAs called short interfering RNAs (siRNAs) that in complex with Ago also initiate the cleavage of complementary endogenous or viral RNAs due to the nuclease activity of Ago. The piRNA-based silencing mechanism is highly conserved and found in all animals, including mosquitoes and other arthropods and is active in a broad range of cell types, including somatic and germline tissues [84–86]. The notable exception is *D. melanogaster*, in which the piRNA system is restricted to the germline. PIWI proteins have expanded in Culicine mosquitoes, while Anophelines retain single *Drosophila* orthologs [86, 87]. In contrast to *D. melanogaster* and Anophelinae, the Culicinae mosquitos are also major vectors of viruses. The negative impact of viruses on the organism is partly prevented by mosquito antiviral siRNAs and piRNAs [86]. The presence of multiple PIWI genes in Culicinae may hint at a history of selection for resistance to the harmful effects of transposon propagation [86, 87].

We might ask why transposons have greatly proliferated during evolution in the Culicine genomes? It has been shown that the short-term effective population size (*N*_*e*_) of *Ae. aegypti* is about 400–600 on average, which is lower than that of many other insects including *Anopheles* species [88]. In comparison, *N*_*e*_ of *An. gambiae* has been estimated to be an order of magnitude greater than the estimates for *Ae. aegypti* [89]. Small *N*_*e*_ accelerates the pace of genetic drift, with a corresponding decrease in the efficacy of selection including purifying selection against TE insertions. In contrast, larger effective population size of Anophelinae facilitated strong positive selection for increased recombination to remove TE insertions and to keep the genome size small. It is possible that Culicines have accumulated transposable elements by chance due to genetic drift in ancestral populations.

Another intriguing possibility is that TE expansion in Culicines is related to the ability of these mosquitoes to transmit a large variety of arboviruses. Mosquitoes combat transposons and viruses with the same Ago and small RNA-based defense systems. Small RNAs generated against viral transcripts might decrease the abundance of small RNAs against transposons in the cellular pool. The integration of viruses into the genomes will lead to the inheritance of the viral genomic fragments and to the change of small RNA repertoire throughout generations, thus affecting the ability of the innate immune system to prevent the transposon expansion. Indeed, a study in *D. melanogaster* demonstrated that viral infections affect transposon transcript levels via modulations of piRNA and siRNA repertoires [90]. The genomes of Culicinae, unlike the Anophelinae genomes, are enriched in endogenous viral elements (EVEs) indicating that integration of viruses into the genomes and changes of small RNA repertoire might have happened continuously in the past. It was also noted that the proportion of small RNAs corresponding to the transposons is relatively low in Culicines in comparison with other dipteran insects. While only 12% of the *D. melanogaster* genome is occupied by transposons [91], almost 18% of small RNAs in their ovaries (mostly piRNAs) correspond to the transposons [86]. In contrast, transposons occupy 58–78% of genomes of *Cx. quinquefasciatus* and *Ae. aegypti*, but only 5–10% of small RNAs were derived from transposons [86, 92]. The loss of transposon-derived small RNAs in Culicine mosquitoes (potentially driven by viral infections) may have permitted transposons to proliferate that led to increase of the genome size during evolution. By contrast, poor ability of Anophelines to transmit arboviruses together with their effective removal TE genomic insertions via recombination have not resulted in expansion of PIWI proteins. Further studies are required to test these hypotheses, but as these would likely require multiple generations over many years these studies linking TE insertions with viral infection may be challenging.

Unlike other insects, mosquitoes have conserved karyotype 2N equal 6 represented by X and Y sex chromosomes and 2 autosomes in the subfamily *Anophelinae* but by 3 autosomes in the subfamily Culicinae, in which male sex is determined by a locus on one of the autosomes, so called homomorphic sex-determining chromosomes (Fig. 6) [93]. This karyotype has been preserved during 260 MYA of mosquito evolution [33]. For comparison, in other insects, such as Hemiptera, the number of chromosomes may significantly vary from 4 to 74 [94]. In *Drosophila* species, the number of chromosomes changes as a result of fusion and fissions of the chromosomal arms whereas the number of chromosomal arms or, so called, Muller elements is preserved between the species [95, 96]. A similar pattern of chromosomal evolution through fusion and fission was shown for Lepidoptera species [97, 98]. In mosquitoes, whole-chromosome fusion and fissions were not detected. Mosquito chromosomal evolution is largely shaped by multiple whole-arm translocations among autosomes (Fig. 6B) and paracentric chromosomal inversions (Fig. 6D, E, Additional file 1: Fig. 10) within chromosomal arms [33, 64, 99]. Although comparison of *An. albimanus* and *An. coluzzii* identified only whole-arm translocations, a comparison of these species with *An. atroparvus* detected partial-arm translocations between the autosomal arms that involve the genetic material of the pericentromeric regions [16]. Our work additionally reveals pericentric inversions between chromosomal arms, including one affecting the homomorphic sex-determining chromosomes in *Cx. quinquefasciatus* and *Ae. aegypti.*

Only one large translocation was identified between one of the arms of chromosome 1 in Culicinae mosquitoes (1p arm in *Cx. quinquefasciatus* or 1q arm in *Ae. aegypti*) and the biggest autosomal arm in Anophelinae mosquitoes e2 (2R arm in *An. coluzzii*) [28, 64]. As a result of this rearrangements, the Anophelinae chromosomal element e2 represents a mosaic of the 1p and 3p arms of *Cx. quinquefasciatus* suggesting two alternative scenarios of chromosomal evolution depending of which karyotype is considered ancestral (Fig. 7). In both scenarios, the ancestral karyotype has homomorphic sex chromosomes as has been demonstrated by the analyses of retro-transposition events [100, 101]. In the first scenario, the ancestral karyotype was similar to the modern karyotype of Culicinae mosquitoes with metacentric homomorphic sex-determining chromosomes 1 (Fig. 7A). In the lineage that led to the ancestor of Anophelinae mosquitoes, the whole arm of the chromosome 1, which has the genetic material of e2, was translocated to one arm of the chromosome 2 creating the largest element in the *Anopheles* karyotype. As a result, the sex-determining chromosome became acrocentric. Later, the joined genetic material was reshuffled by paracentric inversions creating the modern 2R arm in *An. gambiae*. Additionally, one homolog of the acrocentric sex-determining chromosome began to degenerate, eventually becoming the Y chromosome. In the second scenario, the ancestral karyotype was different from the modern karyotypes of both Culicinae and Anophelinae mosquitoes (Fig. 7B). It had acrocentric homomorphic sex chromosomes before the origin of Culicinae and Anophelinae lineages. In the lineage leading to *Anopheles*, one homolog of the acrocentric sex-determining chromosome began to degenerate and became the Y chromosome. No X-autosomal translocations happened in the genus *Anopheles*. In the lineage leading to Culicinae, a part of the genetic material of e2 was translocated from an autosome to the sex-determining chromosome 1. As a result, the sex-determining chromosome 1 became metacentric in Culicine mosquitoes.

Based on the number of rearrangements affecting different chromosomal arms, we compared the rate of chromosomal evolution in different mosquito linages. Our data support previous observation of the more rapid evolution of the X chromosome versus autosomes in *Anophelinae* mosquitoes [33]. In the Culicine lineages, both arms of the homomorphic sex determining chromosome have also evolved faster than the other autosomes but the difference is smaller. Accelerated evolution of the *Drosophila* X chromosome is not as dramatic as that affecting the *Anopheles* X [33], more in line with the difference between homomorphic sex-determining chromosome 1 and other autosomes in *Ae. aegypti*. Interestingly, some groups of insects, including Hemiptera with holocentric chromosomes, show the opposite trend, with sex chromosomes evolving slower than autosomes [94].

## Conclusions

In this study, the integration of advanced genome sequencing technologies with molecular and cytogenetic mapping allowed us to construct a high-quality genome assembly for the vector of West Nile virus *Cx. quinquefasciatus* with 97.67% genome placement to chromosome positions. Genome quality validation using whole genome resequencing and RNA-seq data demonstrated significant improvements in mapping to the new J5 genome versus the previous J3 genome, though some assembly gaps may remain. The comparison of the newly developed genome assembly of *Cx. quinquefasciatus* with the chromosome-scale genome assemblies of the malaria vectors *An. coluzzi* and *An. albimanus* and the arbovirus vector *Ae. aegypti* uncovered new details of mosquito genome evolution. We confirmed a significant expansion of odorant receptor (*OR*) and odorant-binding protein (*OBP*) genes in the *Cx. quinquefasciatus* genome, resulting this species having nearly 50% more *ORs* than *Ae. aegypti* despite having a much smaller genome. Hi-C mapping allowed the identification of a polymorphic chromosomal inversion in *Cx. quinquefasciatus* highlighting the utility of this method in characterizing chromosomal rearrangements in mosquitoes with poor-quality polytene chromosome. Chromosome quotient analysis enabled the approximate location of the male-determining locus, close to the centromere on the p arm of the male-determining chromosome 1. A dramatic expansion of the transposable elements (TEs) was documented in both *Cx. quinquefasciatus* and *Ae. aegypti*. While TEs are concentrated in pericentromeric regions in the genomes of *Anopheles* species, they have spread along the arms toward the telomeres in *Cx. quinquefasciatus* and become evenly distributed along the chromosomes of *Ae. aegypti*. Our work also clarifies details in chromosomal evolution of mosquitoes that included (1) multiple paracentric inversions within chromosomal arms and whole-arm translocations in all mosquito lineages; (2) a large translocation between autosome e2 in Anophelinae and sex-determining chromosome 1 in Culicinae mosquitoes; (3) a pericentric inversion in chromosome 1 of *Cx. quinquefasciatus* and *Ae. aegypti*. Finally, the X chromosome in *An. coluzzii* and the sex-determining chromosome 1 in *Cx. quinquefasciatus* and *Ae. aegypti* have evolved significantly more rapidly than other autosomes in each species. The enhanced understanding of mosquito genome organization generated by this study will guide further efforts to development novel genome-based strategies for the vector control.

## Methods

### Assembly development and annotation

#### Mosquito strain

The Johannesburg (JHB) strain of *Cx. quinquefasciatus* was utilized for the mosquito sequencing. This strain has been previously used for the initial genome sequencing project [28], for the development of the genetic map [34], and for the integration of genetic, genome, and chromosome maps in this species [29]. Later, the initial genome of this strain was improved using Hi-C technology [8].

#### Generation of a paternal TrioCanu assembly

DNA from two parents (mother and father mosquitoes) was sequenced separately using Illumina HiSeqX (100× each) as previously described [17]. Briefly, genomic DNA was isolated using the QiaAMP DNA micro kit (Qiagen, Germantown, MD, USA). Approximately 300 ng of genomic DNA was used to prepare DNA sequencing libraries for each parent following using the NEBNext Ultra II FS DNA Library Prep Kit for Illumina (New England Biolabs, Ipswich, MA, USA). The libraries were sent for sequencing [102]. More than 50 Gb of 2×150 bp reads were obtained from the father and mother. Pooled F1 males from a single pair parent were used as the source for ONT sequencing (Fig. 1B). Genomic DNA was isolated using a Qiagen Genomic Tip DNA Isolation kit (Qiagen, Germantown, MD, USA) similar to previously described method [17]. The purity, approximate size, and concentration of the DNA were tested using a nanodrop spectrophotometer, 0.5% agarose gel electrophoresis, and Qubit dsDNA assay, respectively. Approximately 1μg of DNA was used to generate a sequencing library using the SQK-LSK109 library preparation kit from Oxford Nanopore. Both MinON and PromethION runs were used to obtain approximately 89 Gb of reads. Base calling was performed using either albacore or Guppy [103] with the default filtering setting of Qscore >7. The long ONT sequences from F1 male siblings were successfully separated into paternal (~48.1%) and maternal (~48.9%) reads by TrioCanu according to paternal-specific and maternal-specific k-mers identified from the paternal and maternal Illumina reads (Additional file 2: Table S6). The hemizygotic nature of the M locus ensures that M reads (and reads from a larger M-linked haplotype) are segregated into the paternal fraction. All paternal-specific reads plus unseparated reads are used to obtain a paternal assembly by Canu [39] and then polished using the F0 Illumina paternal reads by three rounds of Pilon [40]. The new assembly was annotated by NCBI via an automated the NCBI Eukaryotic Genome annotation Pipeline [47].

#### Bionano mapping

To perform Bionano mapping ultra-high molecular weight (uHMW) nuclear DNA was isolated from pooled male siblings as previously described [17]. Data collection for optical mapping was performed in a Bionano Saphyr platform as previously described [104]. Molecules were stretched, separated, imaged, and digitized using software installed in a Bionano Genomics Saphyr System and server according to the manufacturer’s recommendations [105]. The molecules were assembled into maps by the Bionano Solve Version 3.3, Tools Version 1.3, RefAligner Version 7989 and Pipeline Version 7981 software. Bionano (BNG) maps were aligned to the described sequence assembly to generate hybrid scaffolds using the Bionano Solve V3.3 suite. The alignment produced only 56 scaffolds, with a total scaffold length of 781.37 Mbp (Additional file 2: Table S7). Sequence was manually curated to trim sequence overlaps, remove secondary contigs due to heterozygosity, and break up mis-assemblies.

#### Hi-C scaffolding

The protocol for in situ Hi-C experiment was described in details elsewhere [106], and it was slightly modified in this study. In brief, ~1000–2000 embryos from JHB colony were collected at the 16–18-h developmental stage. Mosquito eggs were incubated in 70% bleach-water solution for 5 min. After that, they were fixed with paraformaldehyde-based buffer and homogenized with tissue homogenizer, followed by cell lysis. The chromatin was digested by MboI restriction enzyme (New England Biolabs, Ipswich, MA, USA), DNA ends were marked with biotin-14-dATP (Thermo Fisher Scientific, Waltham, MA, USA), and ligated with T4 ligase (New England Biolabs, Ipswich, MA, USA). The crosslinks were reversed, DNA was purified and sonicated with COVARIS M220 (Covaris Limited, Woburn, MA, USA) followed by biotin pull-down and Illumina library preparation. The Hi-C libraries were prepared and sequenced with Illumina HiSeq4000 PE150. Raw Hi-C reads were processed using Juicer protocol to create contact matrices [42]. Then, the 3D-DNA pipeline [8], with J3 genome used as a reference, was applied for developing the draft genome assembly. The draft assembly was visually inspected and reconstructed manually with Juicebox Assembly Durand Tools [107] according to their 3-dimensional contacts. Multiple misassemblies were detected and fixed, haplotigs were identified and removed from the main assembly (Additional file 2: Table S7). The complete Hi-C statistics can be found in Additional file 2: Table S8.

#### Physical mapping

Physical genome mapping was performed using fluorescence in situ hybridization (FISH) on mitotic chromosomes from 4th instar larvae of *Cx. quinquefasciatus* [108]. A total of 143 markers were mapped to chromosomes (Additional file 2: Table S1). DNA from Bacterial Artificial Chromosome (BAC) clones from Notre Dame JHB library [43] were labeled with either Cy3- or Cy5-dUTP (Enzo Life Sciences Inc., Farmingdale, NY, USA) by nick translation and utilized as a DNA probe for FISH. A landmark-based two-color physical mapping approach was utilized for localizing BAC positions in mitotic chromosomes [29, 64]. After FISH chromosomes were stained with YOYO-1 iodide and mounted in a small amount of Prolong Gold antifade reagent under a cover slip (Thermo Fisher Scientific, Waltham, MA, USA). Because chromosome arms in this species are almost equal in size, three molecular landmarks for q arms of each chromosome were used in addition to two BAC clones of interest [29, 64]. Finally, *MyoM* gene and rDNA probes were developed as previously described [29]. Probes were labeled with Cy3- and Cy5-dUTP (Enzo Life Sciences Inc., Farmingdale, NY, USA) and hybridized to the chromosomes [108]. Slides were analyzed using a Zeiss LSM 510 Laser Scanning Microscope (Carl Zeiss Microimaging, Inc., Thornwood, NY, USA) at ×600 magnification.

#### Chromosome quotient analysis

We used CQ [46] to identify the region surrounding the M locus. Pooled females and males from the JHB strain were sequenced respectively for mapping to the J5 assembly. The entire genome was masked using RepeatMasker [109] with the repeat library described in this manuscript. This process was repeated two more times to reduce residual repeat sequences. The masked assembly was split into 1-kb fragments. The fragmented genome was used as the reference for mapping Illumina reads obtained from pooled females and males, respectively. The CQ value for each fragment was calculated by the number of female alignments divided by the number of male alignments (Additional file 2: Table S9). To mitigate the potential bias introduced by a small denominator, fragments showing less than 10 male alignments were excluded from the analysis [21]. We used 0.05 as the cut-off for male-specific fragments [21].

### Assembly and annotation validation

#### Analysis of newly collected shotgun genome sequence data from field specimens

Whole-genome resequencing data from field-caught *Cx. quinquefasciatus* mosquitoes were mapped of the chromosomal contigs of the new (J5) and old (J3) assemblies in order to assess mappability. Ten *Cx. quinquefasciatus* mosquitoes were captured in Santa Barbara (*n*=6) and San Diego (*n*=4), California using Gravid traps, EVS traps, or larval dip collections. DNA was extracted using the NucleoSpin 96 DNA RapidLyse kit (Macherey-Nagel, Germany), and individuals were identified to species via multiplex PCR of the *ACE2* locus [110]. DNA sequencing libraries were prepared using Illumina DNA Prep Kits (Illumina, USA) with dual-unique barcodes. Libraries were then pooled and sequenced at Novogene on an Illumina Novaseq 6000 PE150. Sample metadata, including collection dates and GPS coordinates and individual genome-wide coverage, can be found in Additional file 2: Table S2.

Raw reads were assessed for quality using FastQC [111]. Low-quality bases and adapters were trimmed by Trimmomatic [112]. Trimmed reads were mapped onto the three chromosomal scaffolds of both the J3 and J5 assemblies (excluding extrachromosomal contigs). We used bwa-mem to map the reads with default setting [113] and marked optical and PCR duplicates with Picard MarkDuplicates (Broad Institute, n.d., Cambridge, MA, UK). We calculated coverage after de-duplication using Mosdepth [114] and used the de-duplicated reads for all analyses. First, samtools [115] was used to calculate the fraction of (1) mapped reads, (2) properly mapped paired reads, (3) mapped with MQ>30, and (4) reads whose mates were mapped to different chromosome(s) for each individual and assembly. We further identified reads that did not map to the J3 assembly and tried to map them onto J5. For comparison, we reciprocally identified reads that did not map to J5 and tried to map them onto J3. The re-mapping employed the same pipeline described above, except that any reads with MQ<10 were additionally filtered out. We then calculated the fraction of ‘covered’ bases in non-overlapping 1-Mb windows across each assembly, where covered bases are defined as the fraction of bases within a given window that had at least 1× coverage of reads that did not map to the other assembly. To examine if the fraction of covered bases is impacted by genomic context, fractions of repeats and genic content across the same genomic windows were calculated.

#### RefSeq annotation

Annotation of the new genome assembly of *Cx. quinquefasciatus* was generated for NCBI’s RefSeq dataset [116] using NCBI’s Eukaryotic Genome Annotation Pipeline [117]. The annotation, referred to as NCBI *Culex quinquefasciatus* Annotation Release 100, includes predicted gene models for protein-coding and non-coding genes and pseudogenes and is available from NCBI’s genome FTP site and web resources. Models were predicted using NCBI’s Gnomon algorithm using alignments of transcripts, proteins, and RNA-seq data as evidence. The evidence datasets used for Release 100 are described at [49] and included alignments of available *Cx. quinquefasciatus* mRNAs and ESTs, 2.8 billion RNA-seq reads from 46 SRA runs from a wide range of samples, and RefSeq proteins from *Ae. aegypti* and other insects. The annotation was compared through assembly alignments to the previous annotation provided by the Broad Institute in order to track gene and transcript identifiers and identify merged genes.

#### Analysis of publicly available RNAseq data

To determine if RNA-seq analyses helped to significantly improve the updated assembly, two experimental datasets were analyzed, which consisted of a comparative embryo study (PRJNA454000) and a comparative study of male and female brain (PRJNA612100) that were replicated [51, 52]. Reads were mapped to the current version of the *Cx. quinquefasciatus* genome J5 and the previously available J3 version [8] with STAR using recommended settings [118] to allow for assessing total number of reads that map to specific genes and splice junction identification. Significant differences in expression levels were compared with using DeSeq2 [119]. Genes with significantly different expressions in the updated and old assemblies were compared to establish overlap. Lastly, similarities between the expressional analyses were compared with the use of a Pearson correlation analysis [120] to ensure that the general fold changes are comparable between mapping to this current and the old genome assembly.

#### Gene content and gene family expansion

The genomic annotations and proteomes of *An. albiminus*, *An. coluzzii*, *Ae. aegypti*, and *Cx. quinquefasciatus* J3 and J5 releases were fetched from VectorBase v.57 [121]. Only the longest transcript for each gene was considered for the protein product clustering, the estimation of lengths and number of exons, and counting of merging of gene models. To identify merging of gene models the annotated proteome from one genomic release were searched against the proteome of the other genomic release using diamond (--evalue 1e-5 --masking 0 --outfmt 6 --max-hsps 5 --very-sensitive -k 0 --no-self-hits --id 80 --subject-cover 80) [122], and the fused events were analyzed with mosaicFinder [123]. To analyze the expansion of gene families only orthologs from families having at least one member protein in each of the three mosquito species were considered (9528 proteins of *An. albiminus*, 9827 of *An. coluzzii*, 11,046 of *Ae. aegypti*, and 11,848 of *Cx. quinquefasciatus* J3 and 10,265 *Cx. quinquefasciatus* J5). Each proteome of four mosquito genomic assemblies was clustered with mmseq2 cluster [124] in all-against-all sequence comparisons with length cut-off 70% and identity threshold of 50% (--min-seq-id 0.5 -c 0.7 -s 7.5). In order to focus on multi-gene families, only clusters with more than three members were retained.

### Chemosensory gene annotations and analyses

#### Collection of new RNAseq data from antennae and proboscis

New RNA-seq data was collected from chemosensory tissues of the JHB strain of *Cx. quinquefasciatus* to facilitate the annotation of chemosensory genes. Sharp forceps were used to remove the antennae (pedicel and flagellum) from two groups of ~30 females and two groups of ~30 males, and to remove the distal half of the proboscis from a single group of ~30 females. All animals were reared to 7–9 days post-eclosion, housed in mixed-sex cages, and given access to 10% sucrose solution, but not blood. Dissected tissue was stored at −80°C until RNA extraction with the Zymo Quick-RNA™ MiniPrep Plus Kit. Libraries were prepared using the Illumina® NEBNext® Ultra™ II RNA Library Prep Kit for ultra-low input RNA and sequenced on a NovaSeq 6000 PE150 flow cell. We obtained an average of 28.5Gb data per library with 92.72% of reads having a score >Q30. The quality of raw sequence data was checked using FastQC [111] (default parameters), and adapters were soft clipped using Paired end (PE) trimmomatic [112] (ILLUMINACLIP:TruSeq3-PE.fa:2:30:10:keepBothReads). Reads smaller than 40bp were dropped. HiSat2 [125] was used to align trimmed reads to the three chromosomal contigs of the new assembly with max intron length of 40,000bp. Raw RNAseq data were deposited at NCBI (accession PRJNA939628).

#### Odorant receptor annotations and analyses

Full details on odorant receptor annotations and analyses can be found in the supplement (Additional file 6). Briefly, InsectOR [126] was used to generate first-pass gene models on the three chromosomal scaffolds of the new J5 assembly based on homology to existing *OR* protein annotations from *Ae. aegypti* and *An. gambiae*. The first-pass gene models were then manually refined using new (see above) and previously published RNA-seq data from *Cx. quinquefasciatus* (NCBI accessions PRJNA939628, PRJNA219477) as well as raw tBlastN homology [127] to published mosquito and *Drosophila ORs*. New *OR* annotations were matched to those from the previous assembly [55, 56] using reciprocal blastp searches [127] and inspection of a gene phylogeny that included all *OR* coding sequences from both the old and new annotations, resulting in classification of newly annotated *ORs* as “remain,” “merged,” “new,” or unplaced. A subset of *Cx. quinquefasciatus ORs* with 1-to-1 orthologs in both *Ae. aegypti* and *An. gambiae* were renamed to match the names used in the other species. Metadata, coding sequences, proteins sequences, and full details on *OR* classification and naming can be found in the Additional files (Additional file 2: Table S3, Additional files 3, 4 and 5).

The evolutionary relationships among newly annotated *Cx. quinquefasciatus OR* proteins and those from *Ae. aegypti* and *An. gambiae* were inferred following the methods described in [21], which involved alignment using CLUSTAL Omega [128], trimming using Phylemon2 [129, 130], phylogenetic inference using PhyML v3.0.0 [131], and visualization using Figtree [132]. The expression of newly annotated odorant receptor and odorant-binding protein genes was estimated in key tissues using the new antennal and proboscis RNA-seq data described above as well as previously published data (Additional file 2: Table S5). The new *OR* and *OBP* annotations were merged with the new RefSeq annotation for the J5 assembly using a custom script (Additional file 6). We then used the featureCounts command from the RStudio [133] package Rsubread [134], followed by the DESeq2 package [119] to obtain expression estimates (Additional file 2: Table S10). See Additional file 3 for full details of tree building and expression estimation.

#### Odorant binding protein gene models

*OBP* gene models produced by the RefSeq annotation pipeline using available genomic DNA, protein sequence data from 109 *Cx. quinquefasciatus* odorant binding protein sequences, and antennal RNA-sequencing data were refined manually. Fasta-formatted *OBP* peptide sequences were available from previously published data in Supplemental Fasta Files [59], and raw tblastn homology were used to identify each known *Cx. quinquefasciatus OBP* in the new assembly. Existing gene models from the RefSeq annotation pipeline were named after [59], if tblastn revealed high query coverage > 75% and sequence similarity > 75% and a per exon e-value of 1e−10. Modifications to existing gene models were made according to tblastn results and guidance from spliced antennal RNA-seq reads. Gene models that were missing from the RefSeq annotation, but that could be identified by tblastn homology, antennal RNA-seq alignment, and cysteine residue number and spacing were added manually. For 6 known *OBPs*, no high-quality tblastn hit was found. For these genes, genomic DNA sequences were downloaded from Vectorbase (Release 61, accessed Feb 2023), and blastn homology was used to identify and annotate 2 of these 6 *OBPs.* As was done for *ORs*, when a gene model showed high sequence similarity to 2 proteins, >95% sequence similarity [59], they were merged into a single *OBP* in the new annotation. In some cases, previously undescribed putative *OBPs* were annotated by the RefSeq pipeline. These annotations were confirmed to be *OBPs* and classified into one of the *OBP* subfamilies or subgroups according to the conserved number and spacing of cysteine residues in the peptide sequence [59].

### Comparative genomics of mosquitoes

#### Phylogeny reconstruction

To estimate the phylogeny of mosquitos we have used the assemblies of *An. coluzzi* [18], *An. albimanus* [17], *Ae. aegypti* [21], and current assembly of *Cx. quinquefasciatus* J5. Each assembly was subjected to BUSCO v.4.1.4 [135], analysis to identify the universal single-copy orthologs from OrthoDB (dipteria_odb10 database) [136]. The genomic assembly of *D. melanogaster* (GCF_000001215.4) was also analyzed by BUSCO for further usage as an outgroup. A total of 2842 universal single-copy and non-redundant orthologs, which were present in all four species, were selected for the phylogenetic analysis. The concatenated multiple sequence alignments of the orthologous proteins using MAFFT v7.471 [137] followed by alignment trimming with trimAl v.1.4 [130], (-gt 0.5) resulted in 583,441 amino acid columns that were used to estimate the maximum likelihood species phylogeny using RAxML v.8.0 [138] with the PROTGAMMAJTT model, rooted with *D. melanogaster*. We then used r8s [139] to estimate branch lengths in terms of millions of years with two calibration points: 260 MYA for the common ancestor of mosquitos and *D. melanogaster* and 100 MYA for the common ancestor of *An. albimanus* and *An. coluzzii* [33, 140].

#### Identification of transposable elements and satellites

The identification of low-complexity regions and tandem repeats was performed using *mdust* [141] and Tandem Repeat Finder programs [142]. The genomes masked by satellites and low-complexity regions were further used for the transposable element discovery. The known transposable elements were identified using RepeatMasker (v 4.0.9) [109] with default parameters against the custom non-redundant library prepared from the mosquito RepBase (rb20181026) [61] and TEfam [62] databases. The discovery and annotation of the novel transposable elements was performed with EDTA pipeline (v. 1.9.4) [63]. To generate consensus sequences, the output of the EDTA was manually curated to remove redundancy and false-positive predictions. The manual curation included the removing of the nested and already known transposable elements, and making the non-redundant dataset of the consensus sequences. RepeatMasker was employed for the final annotation the genome sequences, using the consensus sequence of known and newly identified TEs.

#### Identification of tRNA genes among mosquitoes

To identify putative tRNA genes, the genomes of each species were subjected to tRNA-scan 2.0 [143] with setting modified for mosquitoes [144]. An initial minimum quality score of 50 (default is set to 20) was used to obtain high-quality tRNAs that was set to search for eukaryotic tRNA along with displaying an output of structural components of the tRNA. The output of predicted tRNAs from the initial analyses were then vetted through the recommended Eukaryote Confidence Filter under the default conditions as suggested to remove tRNA-derived repetitive elements from tRNAs that function in protein translation [143]. This yielded a high confidence set of tRNA genes for each organism which were considered for comparison.

#### Gene ontology

To identify species-specific orthogroups, annotated proteins from *Ae. aegypti*, *An. albimanus*, *An. coluzzii*, *Cx. quinquefasciatus*, and *D. melanogaster* were analyzed by sequence alignment and phylogenetic orthology inference-based method in OrthoFinder v2 [145]. The number of single-copy orthogroup/orthologous proteins (one-to-one) and co-orthologous and paralogous proteins were identified (one-to-many; many-to-one; many-to-many). Following the identification of species-specific orthogroups, g:Profiler [146] was used to identify enriched gene ontology (GO) categories unique for *Cx. quinquefasciatus*. The enriched GO categories were clustered into functionally related aspects with Revigo [147].

#### Chromosomal rearrangements

To identify single-copy orthologs between pairs of the species, we retrieved protein sequences of the genes and selected the longest isoform for each gene. Then the single-copy orthologs were identified with the OrthoFinder software [145]. The circular plots were produced for each pair of species with Circos software [148] based on the orthology table and species annotation files.

#### The rate of the chromosomal rearrangements

The reconstruction of rearrangements in chromosomal elements was conducted independently for each of them by recreating the architecture of ancestral chromosomal elements followed by their comparison with chromosomes of *An. coluzzi*, *An. albimanus*, *Cx. quinquefasciatus*, and *Ae. aegypti*. A non-redundant set of 2842 single-copy orthologs, which were common for all four analyzed species, was identified from OrthoDB by BUSCO and utilized in this analysis. Since BUSCO does not output the strands on which the orthology genes are located, their orientation in the chromosomes were revealed by mapping back of the protein sequences in the genomes with *tblastn* [149]. The synteny blocks common for all species were identified for each chromosomal elements separately by running first GRIMM_Anchors algorithm to identify non-overlapped anchors (1415 in total for all elements) and then GRIMM_Synteny v.2.0.2 [150] with the requirements of at least two anchors per block (-n) and total gap distance (-G) equal to 750 kb. The calculation of the architecture of ancestral elements was performed with MRG [68] with taking into account the determined topology of species and under the switches “-L,” indicating linearity of chromosomes, and “-H2,” which allows MGR to detect the smallest inversions first. For each element, the determined number of inversions occurred during speciation was normalized to the number of synteny blocks, the size of repeat-masked chromosomal arm (Mb) and divergence time from their last common ancestor (134.6 MYA). Because TEs in the genomes may have their own independent evolutionary trajectories different from non-repetitive parts of the chromosomes, we repeat-masked the genomes for calculating the rate of rearrangements.

### Supplementary Information

**Additional file 1:** **Fig. S1.** Hi-C scaffolding of *Culex quinquefasciatus* genome. **Fig. S2.** tRNA identified from mosquito species. **Fig. S3.** Genome quality validation. **Fig. S4.** Chromosomal locations of *ORs *and* OBPs* annotated in the new assembly. **Fig. S5.** Inferred evolutionary relationships among odorant receptors in *Culex quinquefasciatus*, *Aedes aegypti*, and *Anopheles gambiae*. **Fig. S6.** Odorant receptor (*OR*) expression in adult chemosensory tissues and larvae of *Culex quinquefasciatus*. Expression visualized using the R function pheatmap with the euclidean distance calculation [155]. **Fig. S7.** Odorant-binding protein (*OBP*) expression in adult chemosensory tissues and larvae of *Culex quinquefasciatus*. Expression visualized using the R function pheatmap with the euclidean distance calculation [155]. **Fig. S8.** Genome landscape in*Culexquinquefasciatus***. Fig. S9.**Evolution of transposable elements in mosquitoes. **Fig. S10.** Gene order reshuffling in mosquito chromosomes.

**Additional file 2:** **Table S1.** A list of the BAC clones mapped by FISH to the chromosomes of *Culex quinquefasciatus*. **Table S2.** Genome Resequencing Sample Metadata. **Table S3.** Odorant Receptor Annotation Details. **Table S4.** Novel odorant binding proteins and their subfamily/group classifications in *Culex quinquefasciatus*. **Table S5.** Chemosensory Gene Expression Sample Metadata. **Table S6.** Statistics related to TrioCanu separation of the maternal and paternal reads. **Table S7.** Assembly statistics through the process steps. **Table S8.** Hi-C Statistics. **Table S9.** QC and mapping information on the male and female Illumina reads used for CQ analysis. **Table S10.** Log Transformed FKPM Chemosensory Gene Expression.

**Additional file 3.** Odorant Receptor coding sequences from *Culex quinquefasciatus.*

**Additional file 4.** Odorant Receptor proteins from *Culex quinquefasciatus.*

**Additional file 5.** Odorant Receptor and Odorant Binding Protein Annotations.

**Additional file 6.** Detailed Methods for Odorant Receptor Annotations.

## Data Availability

The genome assembly of the JHB strain of *Cx. quinquefasciatus* VPISU-Cqui_1.0_pri_paternal J5 is available at NCBI website (NCBI) under accession number GCF_015732765.1  [38]. The draft Canu assembly of the genome can be found at Culex quinquefasciatus JHB Genome assembly using Flye https://datadryad.org/stash/share/Zr4xg0TuUXNozoarBqjYQD2cDIF3eni71jqdrPMyr08; Doi:10.5061/dryad.rv15dv4cs [57]. Raw Hi-C data of JHB strain of *Cx. quinquefasciatus* can be found at NCBI under accession number PRJNA665323 [151]. Raw RNAseq data from adult antennae and proboscis are available at NCBI under accession number PRJNA939628 [152]. Raw genome resequencing data from field-collected individuals are available at NCBI under accession number PRJNA980724 [153]. The canonical sequences of identified of TEs for *Ae. aegypti*, *An. coluzzii*, *An. gambiae*, and *Cx. quinquefasciatus* will be available at FigShare https://figshare.com/s/1f6a69f78f20107734e0, doi: 10.6084/m9.figshare.23337092 upon the publication [154].

## Abbreviations

BAC: Bacterial Artificial Chromosome
BLASTp: Basic Local Alignment Search Tool, protein
BUSCO: Benchmarking Universal Single-Copy Orthologs
CQ: Chromosome quotient
EVEs: Endogenous viral elements
FISH: Fluorescence in situ hybridization
GO: Gene Ontology
JHB: Johannesburg
LINE: Long Interspersed Nuclear Elements
LTR: Long Terminal Repeats
MGR: Multiple Genome Rearrangements
MITEs: Miniature Inverted-repeat Transposable Elements
MYA: Million years ago
N50: The length of the shortest read in the group of longest sequences that together represent at least 50% of the nucleotides in the set of sequences
*OBPs*: Odorant-binding proteins
*N*_*e*_: Effective population size
*ORs*: Odorant receptors
ONT: Oxford Nanopore Technology
PacBio: Pacific Biosciences
PE: Paired end
piRNAs: Piwi-interacting RNAs
QV: Quality value
siRNAs: Short interfering RNAs
TEs: Transposable elements
TIRs: Terminal Inverted repeats
uHMW: Ultra-high molecular weight
UTRs: Untranslated regions
