# Supplementary material for: The chromosome-scale genome assembly for the West Nile vector Culex quinquefasciatus uncovers patterns of genome evolution in mosquitoes
Source: BMC Biol. 2024 Jan 25;22:16. doi: 10.1186/s12915-024-01825-0 (PMC10809549; doi:10.1186/s12915-024-01825-0)
Supplement: Supplementary file 1 — Additional file 1: Fig. S1. Hi-C scaffolding of Culex quinquefasciatus genome. Fig. S2. tRNA identified from mosquito species. Fig. S3. Genome quality validation. Fig. S4. Chromosomal locations of ORs and OBPs annotated in the new assembly. Fig. S5. Inferred evolutionary relationships among odorant receptors in Culex quinquefasciatus, Aedes aegypti, and Anopheles gambiae. Fig. S6. Odorant receptor (OR) expression in adult chemosensory tissues and larvae of Culex quinquefasciatus. Expression visualized using the R function pheatmap with the euclidean distance calculation [155]. Fig. S7. Odorant-binding protein (OBP) expression in adult chemosensory tissues and larvae of Culex quinquefasciatus. Expression visualized using the R function pheatmap with the euclidean distance calculation [155]. Fig. S8. Genome landscape inCulexquinquefasciatus. Fig. S9.Evolution of transposable elements in mosquitoes. Fig. S10. Gene order reshuffling in mosquito chromosomes. [file 12915_2024_1825_MOESM1_ESM.pdf]

Additional File 1. Figures S1 – S10.

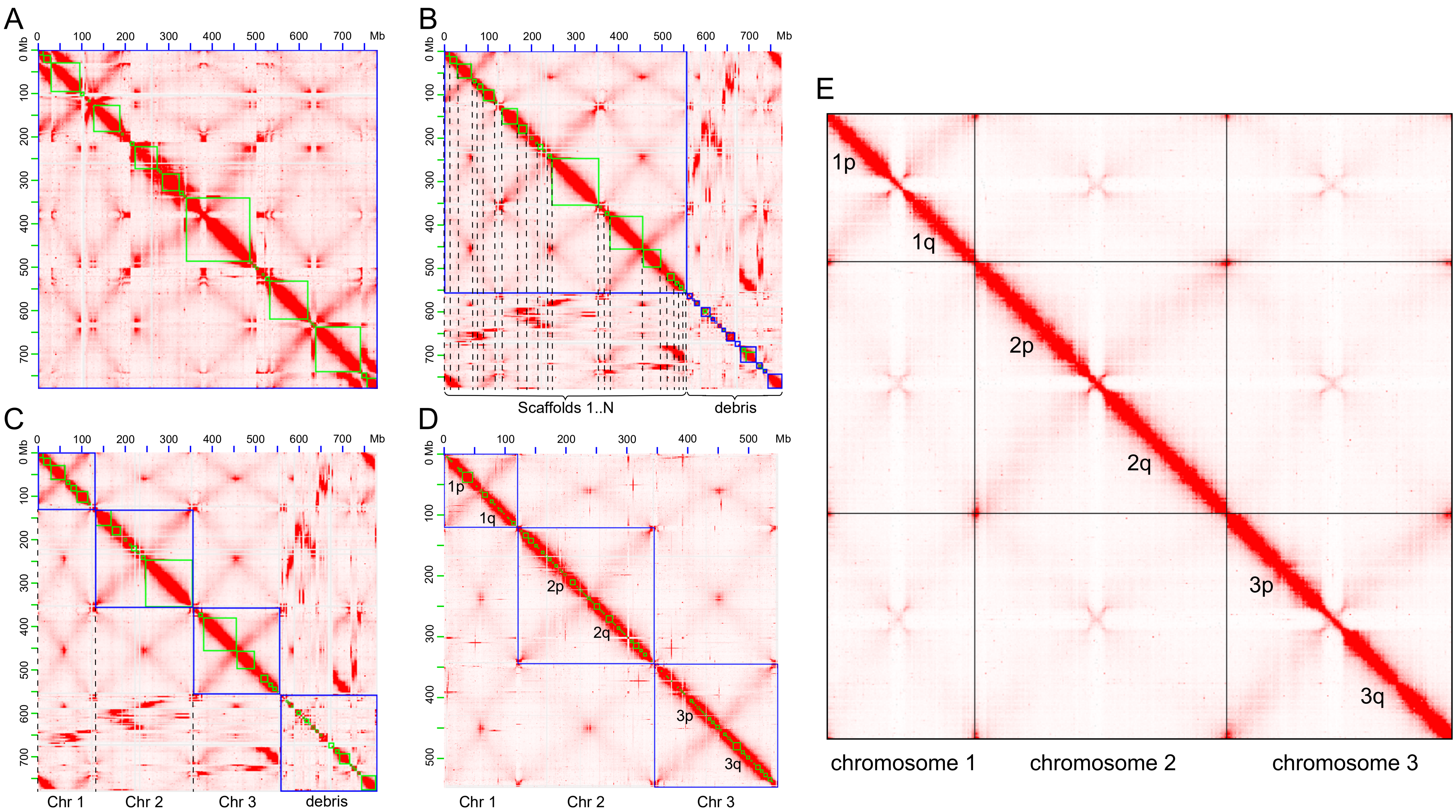

Fig. S1. Hi-C scaffolding of *Culex quinquefasciatus* genome. The figure represents the process of genome assembly scaffolding with Juicebox tools. **A.** The first draft genome assembly generated by 3D-DNA application. **B.** Removal of misassemblies and haplotigs. **C.** Segregation of the assembly into three chromosomes and debris. **D.** The heat map after finalizing chromosomal arm orientation by comparison with the physical map (debris were removed). **E.** The final genome assembly polished with ONT and Bionano mapping.

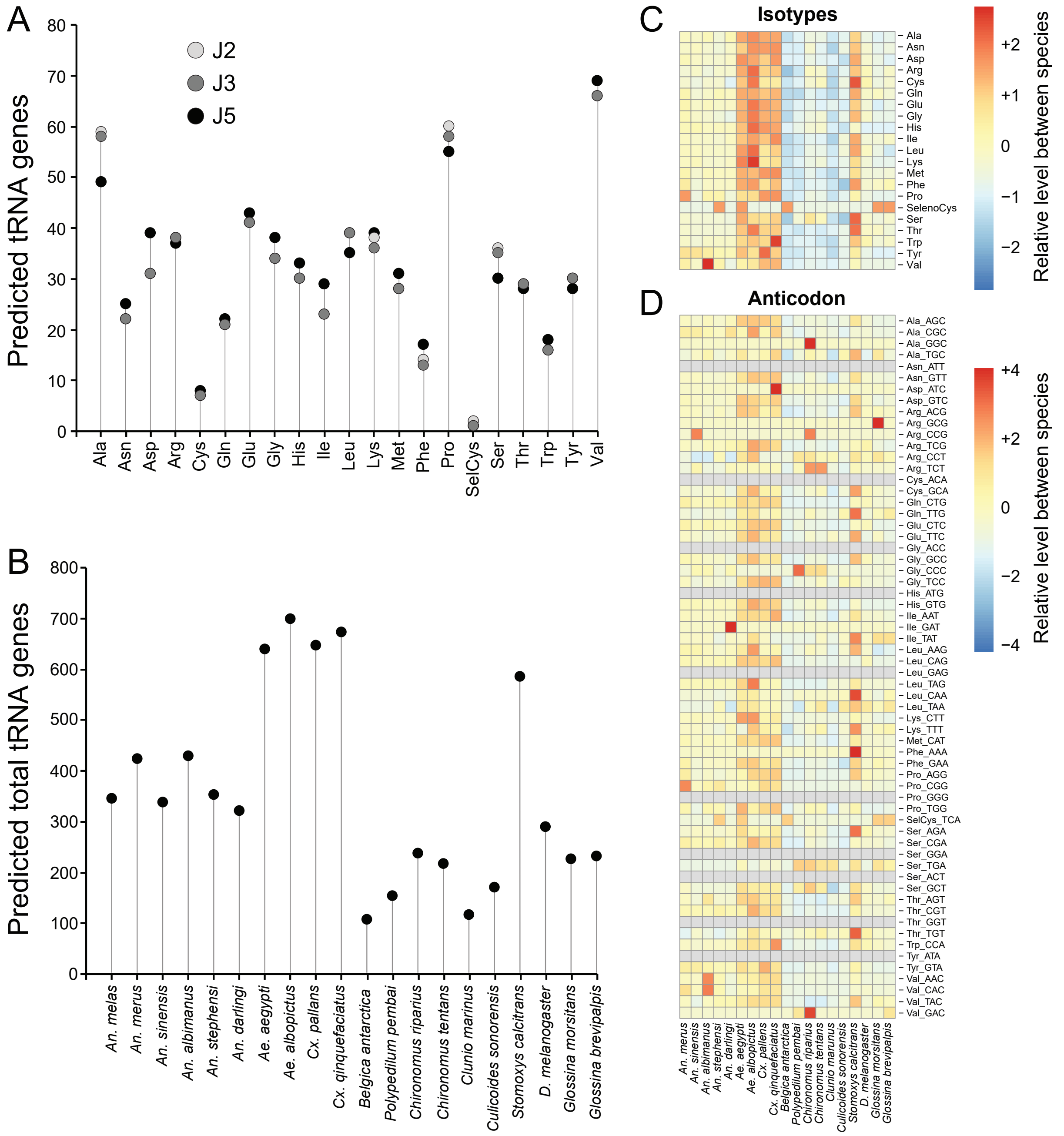

Fig. S2. tRNA identified from mosquito species. A. Changes in the predicted tRNA genes associated with specific amino acids between J2 in light gray [29], J3 in dark gray [9], and current genome assembly J5 in black. **B.** Predicted tRNAs among Culicinae when compared to Anophelinae and other closely related dipterans. **C.** Differences in tRNA isotypes between among Culicinae when compared to Anophelinae and other closely related dipterans. **D.** Differences in tRNA anticodons between among Culicinae species when compared to Anophelinae and other closely related dipterans. Relative levels are the differences in tRNA gene numbers between the species across the rows where the average for all species is set as zero. Gray color indicates no differences in number across species.

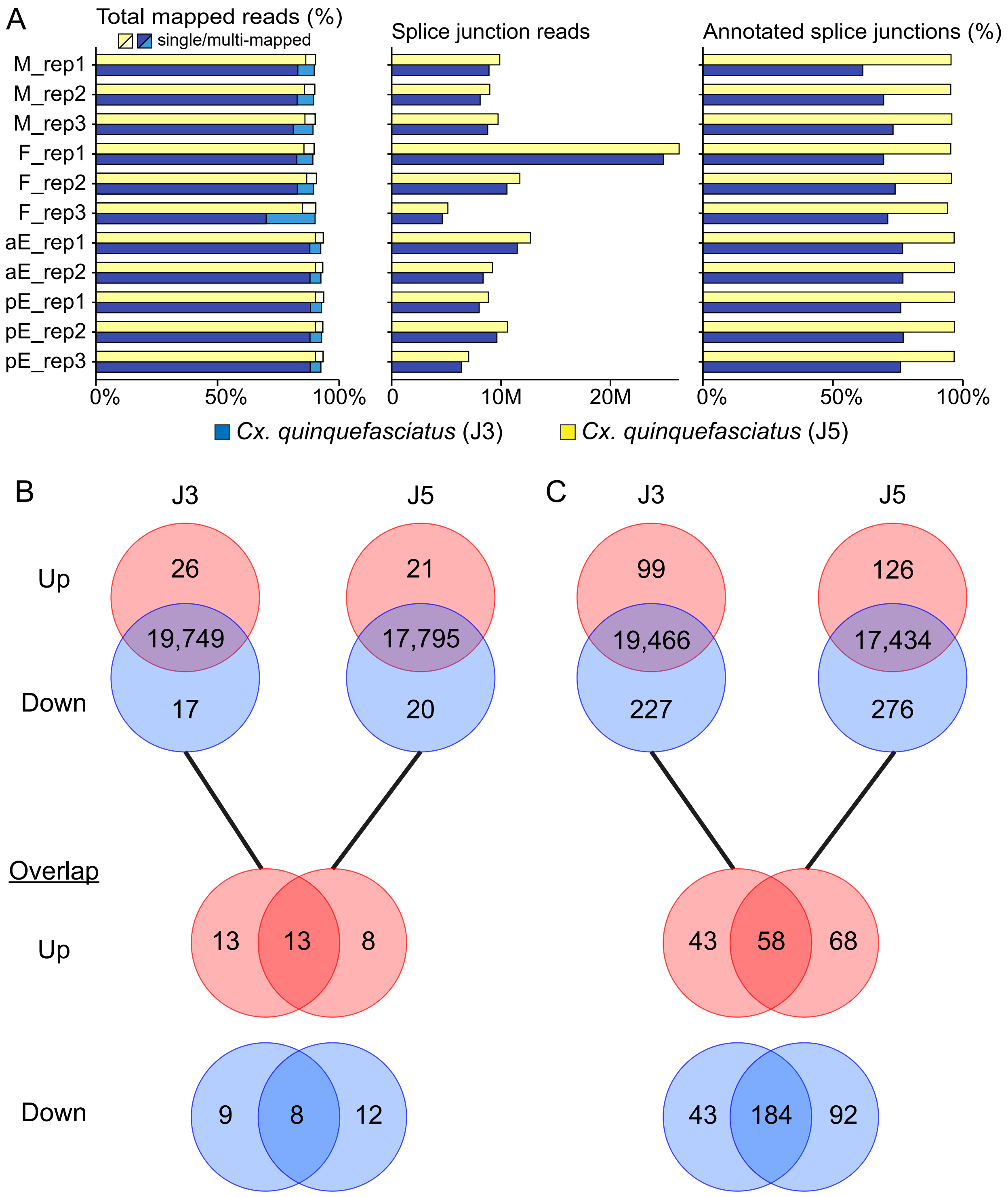

Fig. S3. Genome quality validation. A. The mapping of RNA-seq data from embryo [52] and male and female brain [53] to the current J5 (shown in yellow) and previous J3 [9] (shown in blue) genome assemblies of *Culex quinquefasciatus*. From left to right: the proportions of single-mapped and mulitple-mapped reads mapped on the protein-coding genes annotated for the corresponded genome assembly; the number of reads on splice junctions; the proportions of the annotated splice junctions, are shown. Samples are indicated as follows: M – male brains, F – female brains, pE – posterior poles of the embryos, aE – anterior poles of the embryos. For each sample from 2 to 3 biological replicates (rep) are shown. **B–C.** A comparison of the differentially expressed genes from two RNA-seq projects between J2 [29]/J3 [9] and current assembly J5 of *Culex quinquefasciatus*. Significantly different transcript levels for genes from the anterior and posterior of embryos from [52] (**B**). See also Fig. 3D. Significantly different transcript levels for genes from male and female brains from [53] (**C**). Top is the individual analyses for J3 and J5 assemblies. Bottom is the overlap between the two analyses. Significance was set with the adjusted P value of 0.05.

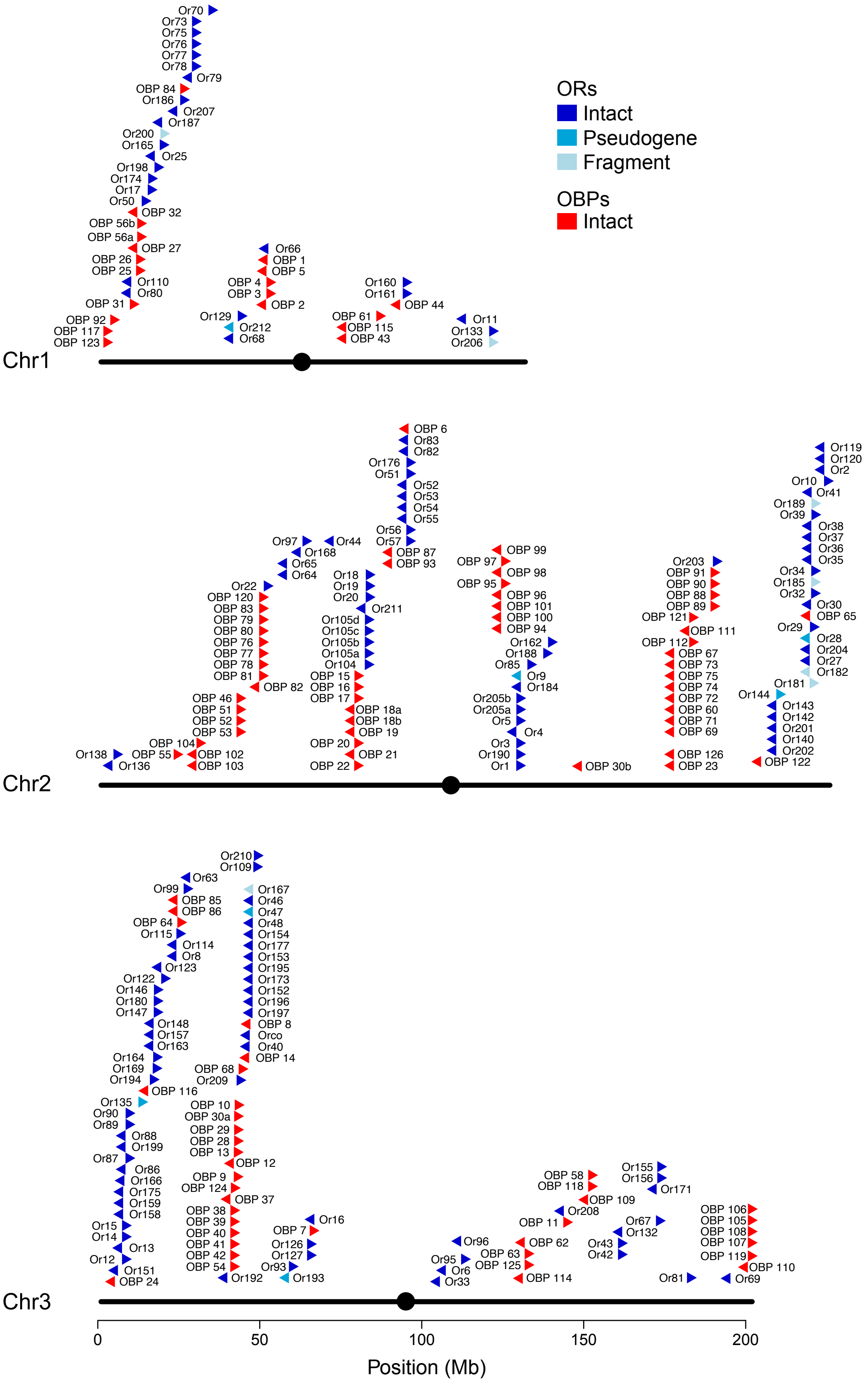

Fig. S4. Chromosomal locations of *ORs* and *OBPs* annotated in the new assembly. Arrowhead position/direction indicates the location/strand of each *OR* or *OBP* along the three chromosomes of J5. Black circles along chromosomes mark the approximate location of centromeres. No *OBP* pseudogenes or fragments were identified in the new assembly.

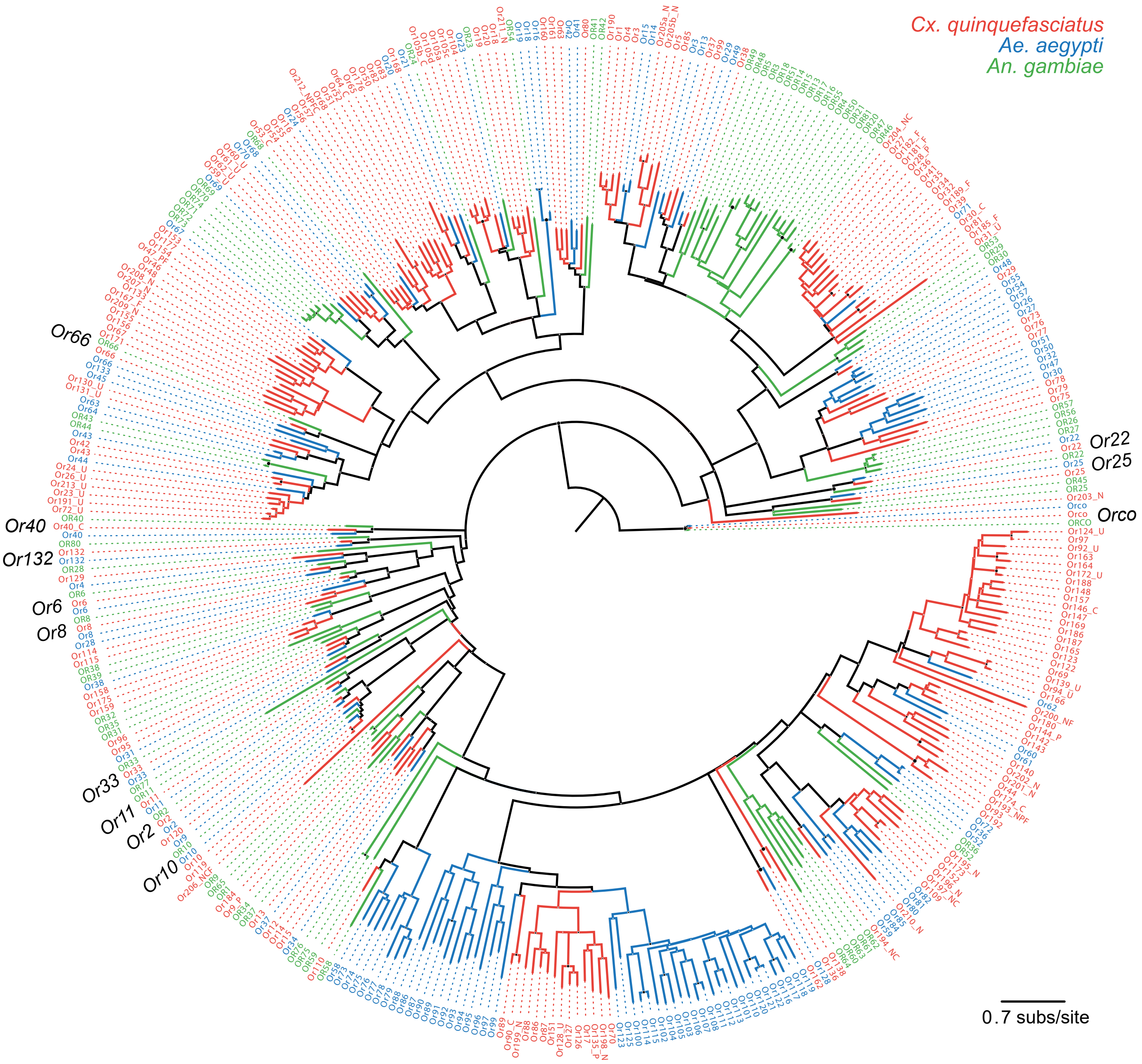

Fig. S5. Inferred evolutionary relationships among odorant receptors in *Culex quinquefasciatus*, *Aedes aegypti*, and *Anopheles gambiae*. Maximum likelihood tree inferred using PhyML v3.0.0, with the size of black circles indicating the relative confidence of a given node (according to approximate likelihood ratio tests). The names of OR groups with a single conserved ortholog in each of the three mosquitoes are highlighted in black around the perimeter of the tree (note that *Cx. quinquefasciatus* orthologs were renamed to match the other species). Suffixes after *Cx. quinquefasciatus* protein names are as follows: U, unplaced; N, new; P, pseudogene; F, fragment; C, corrected (see detailed methods in Additional File 4 and Additional File 2: Table S3).

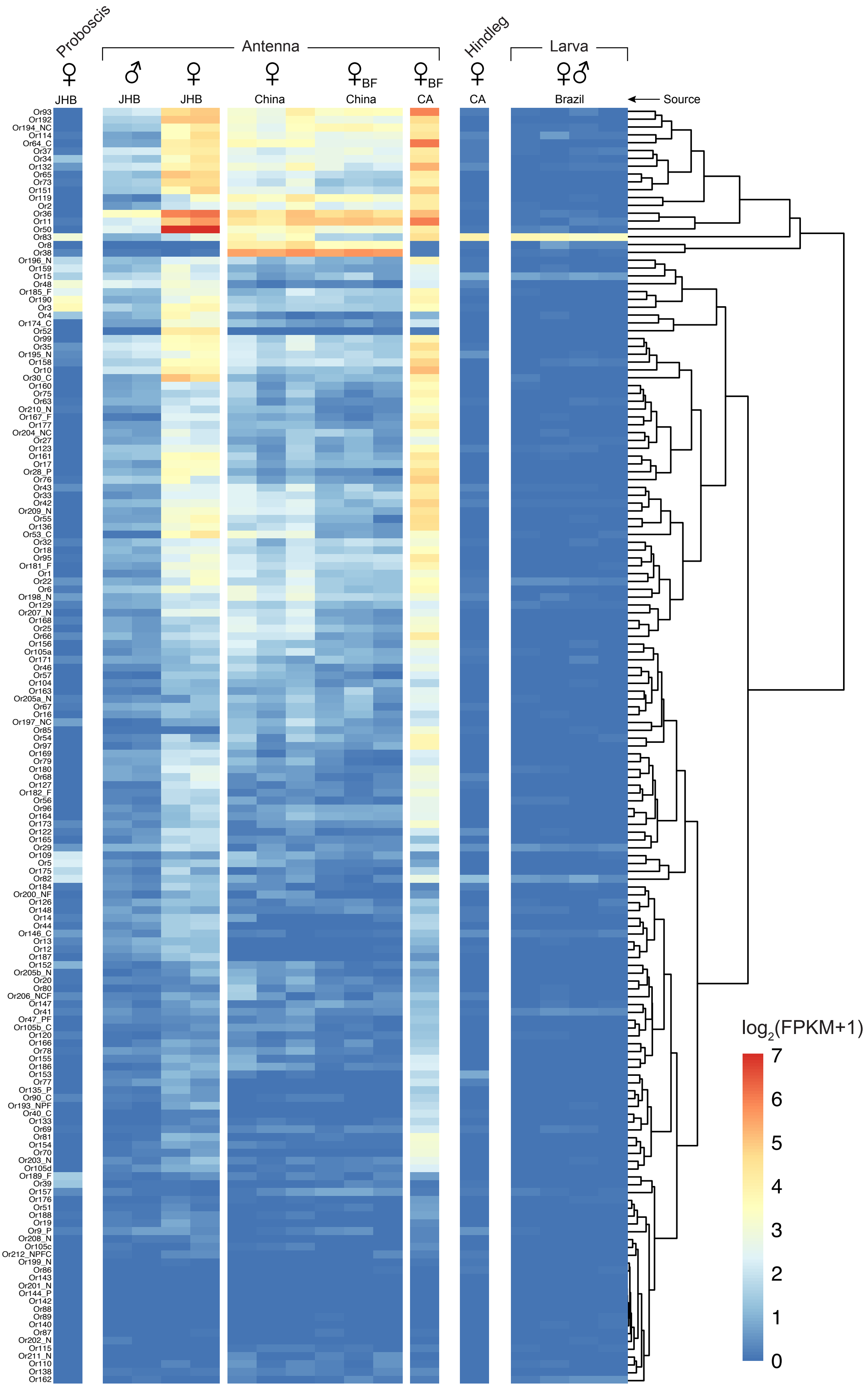

Fig. S6. Odorant receptor (*OR*) expression in adult chemosensory tissues and larvae of *Culex quinquefasciatus*. New and previously published bulk-tissue RNAseq data were used to estimate *OR* expression based on the new genome assembly and *OR* annotations. Expression was quantified using the fpkm function in DESeq2 [118] and visualized using the R function *pheatmap* with the euclidean distance calculation [156]. Raw expression estimates are provided in Additional File 2: Table S5.

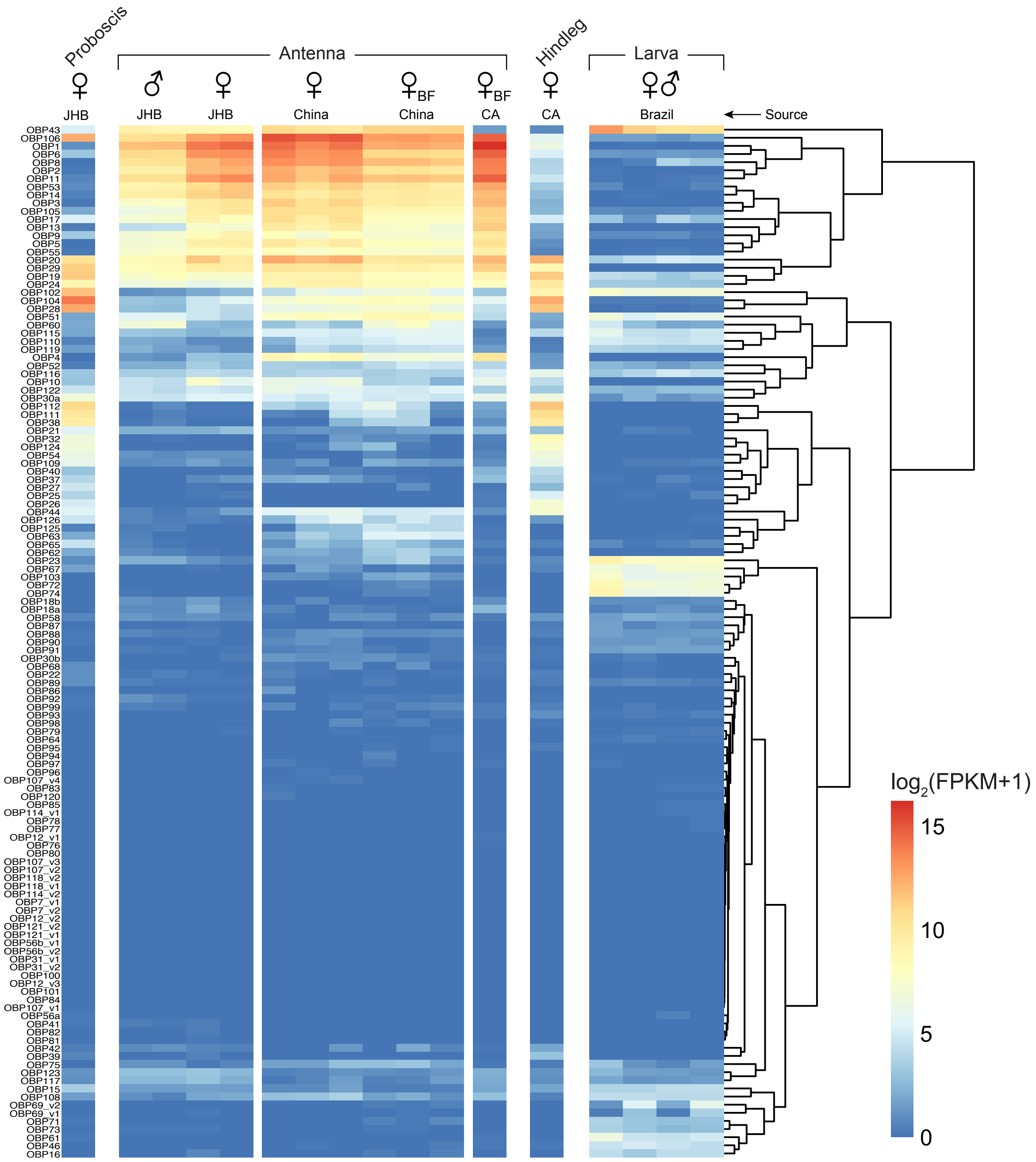

Fig. S7. Odorant-binding protein (*OBP*) expression in adult chemosensory tissues and larvae of *Culex quinquefasciatus*. New and previously published bulk-tissue RNAseq data were used to estimate *OBP* expression based on the new genome assembly and *OBP* annotations. Expression was quantified using the fpkm function in DESeq2 [118] and visualized using the R function *pheatmap* with the euclidean distance calculation [156]. Raw expression estimates are provided in Additional File 2: Table S5.

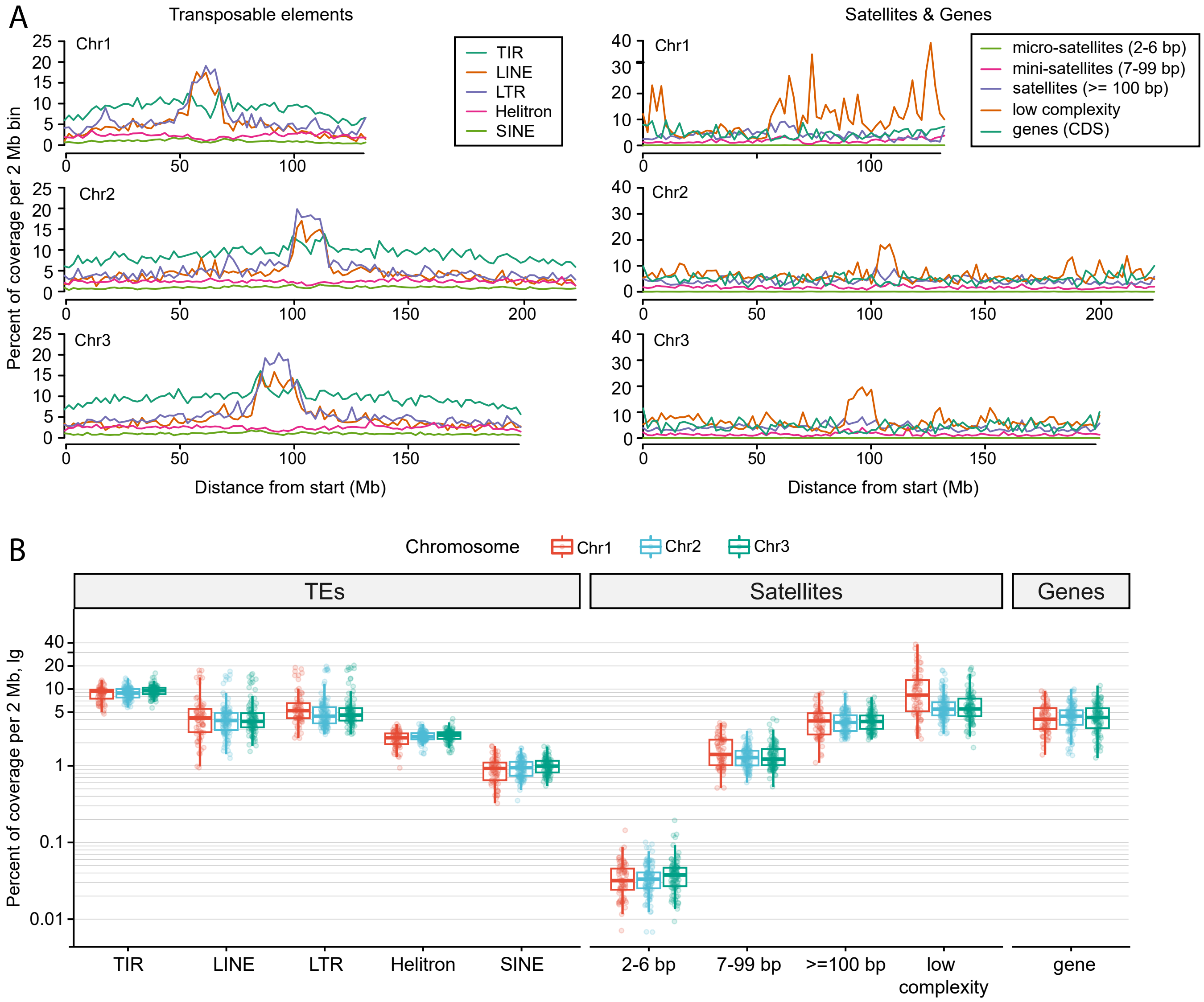

Fig. S8. Genome landscape in *Culex quinquefasciatus*. A. The profiles of coverage of the transposable elements, the satellites, low-complexity regions, and genes along the chromosomes. Each chromosome was split into 2 Mb bins with the following calculations of the number of bp occupied by the genetic elements. Different genomic features are indicated by different colors. **B.** The boxplot with the comparison of the coverage of chromosomes by the transposable elements, the satellites, and genes. The coverage was determined for each 2 Mb bin. Chromosomes are indicated by different colors.

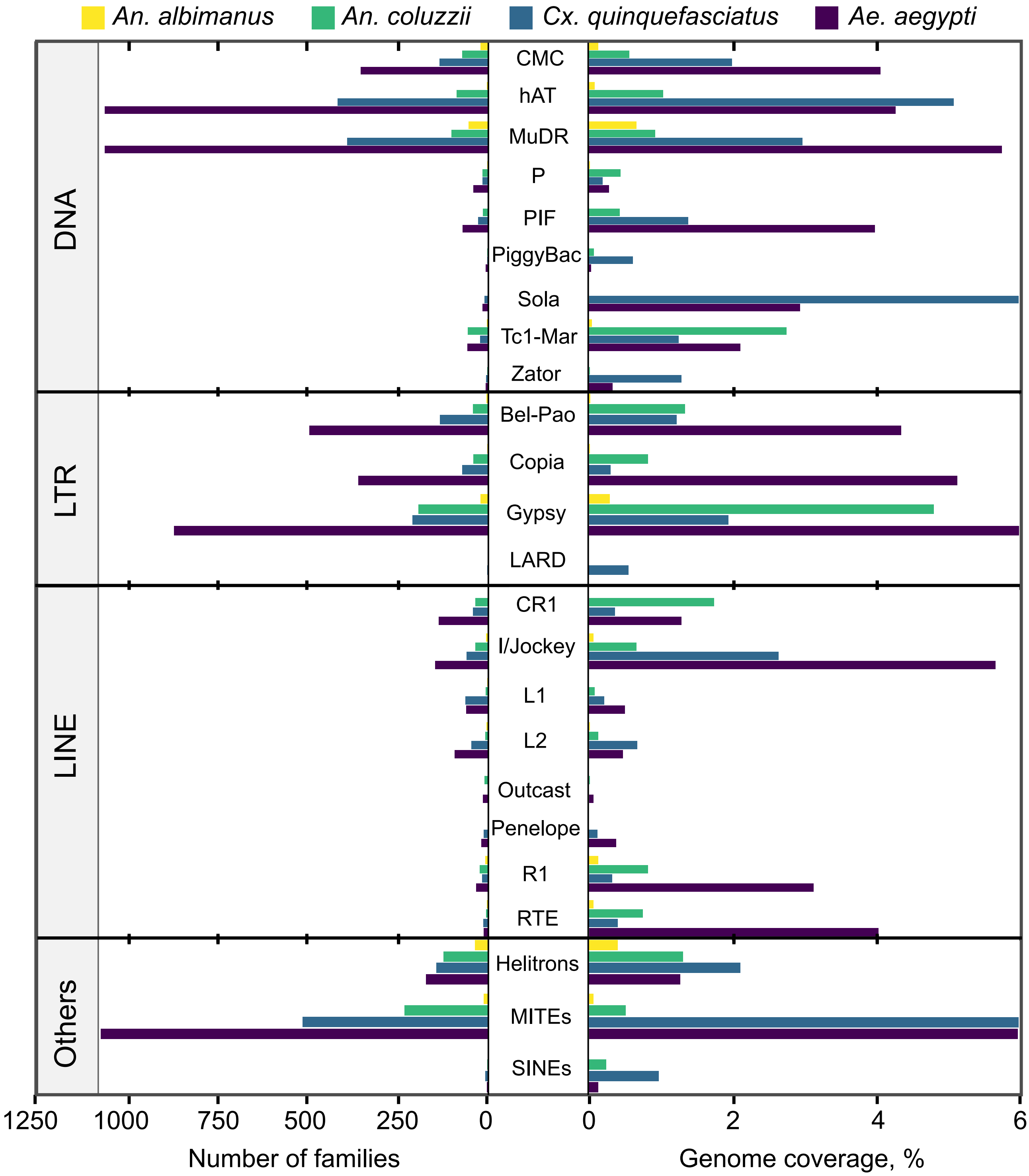

Fig. S9. Evolution of transposable elements in mosquitoes. The number of families and the content (coverage, %) of the identified transposable elements in the genomic assemblies of four mosquito species.

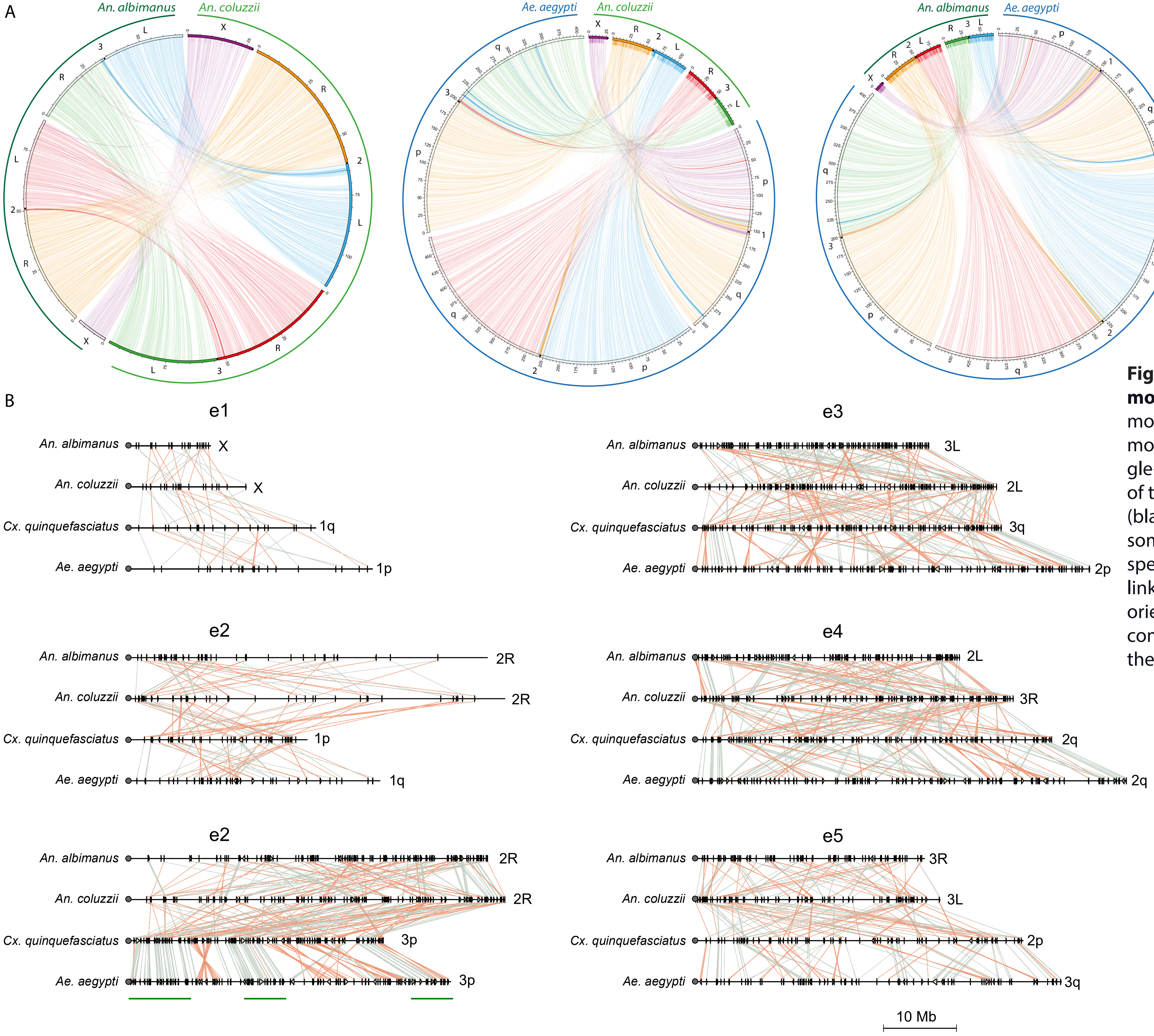

Fig. S10. Gene order reshuffling in mosquito chromosomes. A. Chromosomal syntheny plots between mosquito species based on the single-copy orthologs. **B.** The location of the identified syntenic blocks (black rectangles) in the chromosomal elements for four mosquito species. The syntenic blocks are linked by dark green lines if their orientation is the same in two compared species, or orange lines if their orientation was reversed.
