# Supplementary material for: The chromosome-scale genome assembly for the West Nile vector Culex quinquefasciatus uncovers patterns of genome evolution in mosquitoes
Source: BMC Biol. 2024 Jan 25;22:16. doi: 10.1186/s12915-024-01825-0 (PMC10809549; doi:10.1186/s12915-024-01825-0)
Supplement: Supplementary file 6 — Additional file 6. Detailed Methods for Odorant Receptor Annotations. [file 12915_2024_1825_MOESM6_ESM.docx]

**SUPPLEMENTARY MATERIAL**

**Detailed methods for odorant receptor annotations and analyses**

***Generating gene models.*** We used InsectOR [1] to generate first-pass gene models on the three chromosomal scaffolds of the new assembly based on existing OR protein annotations from both *Ae. aegypti* and *An. gambiae* using an alignment cluster cutoff of 2 and completion cutoff of 300, where all alignment options were selected except for HMMTOP. OR proteins for *An. gambiae* were retrieved by filtering the vectorbase annotation of AgamP4.12 [2] for genes with ‘odorant receptor’ in their description. OR proteins for *Ae. aegypti* were taken directly from Supplementary Data 20 in [3] Since a few *Aaeg* ORs have large introns, we ran exonerate with max intron boundaries of both 40,000 and 1,000,000 and provided both outputs to the InsectOR pipeline.

We manually refined the first-pass gene models from InsectOR in WebApollo based on new (NCBI accession PRJNA939628) and previously published (NCBI accession PRJNA219477) RNAseq data as well as raw tBlastN homology (e-value cutoff 1e-10) [4] to published *Ae. aegypti*, *An. gambiae*, and *D. melanogaster* ORs. *D. melanogaster* ORs were retrieved by filtering from the NCBI assembly of “6 plus ISO1 MT” [5] for genes with ‘odorant receptor’ in their description. Once existing InsectOR models had been manually refined, we conducted another round of tBlastN using the new protein models as well as previous *Cx. quinquefasciatus* protein sequences [6,7] in order to catch additional *Cx. quinquefasciatus*-specific ORs. Anytime our BLAST searches uncovered hits that did not overlap with an existing gene model, we used genewise [8] (default parameters with flags -gff -both) to generate a new gene model in the region (±500,000bp from tBlastN hit) based on homology to the best blast query.

Gene models encoding proteins <150 amino acids long were ultimately discarded and those 150-350 amino acids long were labeled fragments (Supplementary Table 3 column F). In 13 cases, we ‘corrected’ loss-of-function (LOF) mutations present in the genome assembly based on available RNA sequence data showing a small insertion, deletion, or substitution capable of preserving the coding sequence (Supplementary Table 3 columns I-K). However, 4 full-length annotations (non-fragments) had LOF mutations that could not be corrected based on available sequence data and were therefore labeled pseudogenes (Supplementary Table 3, columns G-H).

We initially matched our new OR annotations to those from the previous assembly [6,7] using reciprocal blastp searches [4]. We found that a search for reciprocal best hits with an E-value cutoff of 1e-10 yielded clear 1-to-1 matches for most genes. However, some best matches showed lower than expected amino acid sequence identity (<95%), while other genes had one-to-many relationships between assemblies. For this reason, we manually matched genes between assemblies by inspecting a tree of new and pre-existing protein sequences: protein sequences were aligned using CLUSTAL Omega [9], alignments trimmed with TrimAl with the “gappyout” option, and the phylogeny was constructed using PhyML [10]. Each new annotation was labelled as ‘remain’, ‘merged’, or ‘new’ depending on whether it was paired in the tree with a single OR from the previous annotation (remain), 2–3 closely related ORs from the previous assembly (all with >95% sequence identity) (merged), or no homologous OR found on the chromosomal contigs of the J3 assembly (new) (Supplementary Table 3, column B). The 24 ‘new’ ORs were furthered divided into three groups: they were classified as ‘newly assembled’ in cases where the gene was annotated in the J3 assembly but found on an extrachromosomal contig, ‘newly annotated’ in cases where the gene was present on either the chromosomal or extrachromosomal contigs of J3 but not annotated, or ‘newly present’ in cases where the gene could not be found anywhere in the J3 assembly (Supplementary Table 3, columns C-E). Conversely, a small group of previously annotated ORs did not have any clear match in the new assembly (n=19). About half of these were present on the alternative haplotypes of the new assembly (Supplementary Table 3, column U) and all of them were present in the raw oxford nanopore data used to generate the new assembly (data not shown). We therefore consider these genes to be real, but ‘unplaced’ in the new assembly (Supplementary Table 3, column B).

In most cases, we named newly annotated ORs after the corresponding genes in the previous annotation. However, we chose to rename 11 ORs to match their conserved 1-to-1 orthologs in *Ae. aegypti* and *An. gambiae* (Supplementary Table 3, columns L-O)*.* The *Aedes* and *Anopheles* orthologs already shared the same name in all but one case, but the *Cx. quinquefasciatus* copies were initially given unrelated names, obscuring homology. In order to rename these 11 conserved ORs we had to ‘steal’ the relevant integer names from other, unrelated ORs in the *Cx. quinquefasciatus* annotation, and thus were also forced to rename those unrelated genes. For example, the conserved ortholog of *AaegOr2* and *AgamOr2* was previously named *CquiOr121*. In order to rename this gene *CquiOr2*, we had to additionally rename the OR that was formerly called *CquiOr2* (now *CquiOr190*). To minimize confusion, we list and explain all name changes in Supplementary Table 3, columns L-O. In some cases, we also list previous studies that address the function, expression, or sequence of the given receptors using their old names. Genes that gave up their names to any of these 11 conserved orthologs, as well as ‘newly annotated’ and ‘newly present’ genes were given the next available integer names between 184 and 212. Metadata, coding sequences, proteins sequences, and annotation details for all ORs can be found in the Supplementary Material (Supplementary Table 3, metadata; Supplementary Data File 1, coding sequences; Supplementary Data File 2, protein sequences, Supplementary Data File 3, annotation gff).

***Tree building.*** We inferred the evolutionary relationships among newly annotated *Cx. quinquefasciatus* OR proteins and those from *Ae. aegypti* and *An. gambiae* following the methods described in [3]. OR protein sequences for *An. gambiae* were obtained from the authors of [11], while those for *Ae. aegypti* were retrieved from [3]. OR proteins were aligned using CLUSTAL Omega through the EMBL-EBI portal with default values [9]. Premature stop codons and frameshift mutations were removed from coding sequences before translation and alignment for all pseudogenes and fragments (corrected sequences provided in Supplementary Data File 2). Phylemon2 then took the aligned sequences and trimmed them using gappyout from TrimAl v1.3 with default options [12,13]. The tree was generated using the trimmed alignment file and PhyML v3.0.0 with default options except for selecting “Amino Acid” data type and “Best of NNI and SPR search” [10]. Figtree [14] was used to visualize the tree, which was then rerooted using the small clade of *Orco* orthologs.

***Estimation of OR and OBP expression.*** We estimated the expression of ORs and OBPs using our antennae and proboscis RNAseq data as well as previously published data available in the NCBI-SRA (Supplementary Table 4). A custom script was written using awk [15] and bedtools [16] to merge the new OR and OBP annotation gff with the RefSeq annotation for the new assembly (Supplementary Data File 3). We then used the featureCounts command from the RStudio [17] package Rsubread [18] to quantify the number of reads aligned to the exons of each gene (GTF.featureType=”exon”, GTF.attrType=“transcript_id”, ignoreDup=TRUE, allowMultiOverlap=FALSE, autosort=TRUE, set countMultiMappingReads=FALSE). We ran featureCounts twice, first with isPairedEnd set to TRUE on the reads that remained paired after trimming and then with isPairedEnd set to FALSE on the reads that lost their pair after trimming. We then merged the resulting matrices and used the DESeqDataSetFromMatrix function from the DESeq2 package [19] with a source design to control for batch effects from data origin. Finally, we used the fpkm function in DESeq2 with robust set to TRUE to obtain expression estimates for plotting. The fpkm function uses the median ratio method to normalize by library size and calculates fragments per kilobase per million mapped fragments to normalize by gene length. Gene lengths for fpkm normalization were retrieved from the output of the featureCounts list object. The fpkm normalized reads were transformed by log2(FPKM+1) (Supplementary Table 5), and plotted using the pheatmap function from the pheatmap library [20].

**REFERENCES CITED IN SUPPLEMENTARY MATERIAL**

1. Karpe SD, Tiwari V, Ramanathan S. InsectOR—Webserver for sensitive identification of insect olfactory receptor genes from non-model genomes. PLOS ONE. 2021;16:e0245324.

2. Giraldo-Calderón GI, Harb OS, Kelly SA, Rund SS, Roos DS, McDowell MA. VectorBase.org updates: bioinformatic resources for invertebrate vectors of human pathogens and related organisms. Curr Opin Insect Sci. 2022;50:100860.

3. Matthews BJ, Dudchenko O, Kingan SB, Koren S, Antoshechkin I, Crawford JE, et al. Improved reference genome of Aedes aegypti informs arbovirus vector control. Nature. 2018;563:501–7.

4. Ye J, McGinnis S, Madden TL. BLAST: improvements for better sequence analysis. Nucleic Acids Res. 2006;34:W6–9.

5. Sayers EW, Bolton EE, Brister JR, Canese K, Chan J, Comeau DC, et al. Database resources of the national center for biotechnology information. Nucleic Acids Res. 2022;50:D20–6.

6. Leal WS, Choo Y-M, Xu P, da Silva CSB, Ueira-Vieira C. Differential expression of olfactory genes in the southern house mosquito and insights into unique odorant receptor gene isoforms. Proc Natl Acad Sci. 2013;110:18704–9.

7. Taparia T, Ignell R, Hill SR. Blood meal induced regulation of the chemosensory gene repertoire in the southern house mosquito. BMC Genomics. 2017;18:393.

8. Birney E, Clamp M, Durbin R. GeneWise and Genomewise. Genome Res. 2004;14:988–95.

9. Sievers F, Wilm A, Dineen D, Gibson TJ, Karplus K, Li W, et al. Fast, scalable generation of high-quality protein multiple sequence alignments using Clustal Omega. Mol Syst Biol. 2011;7:539.

10. Guindon S, Dufayard J-F, Lefort V, Anisimova M, Hordijk W, Gascuel O. New Algorithms and Methods to Estimate Maximum-Likelihood Phylogenies: Assessing the Performance of PhyML 3.0. Syst Biol. 2010;59:307–21.

11. Neafsey DE, Waterhouse RM, Abai MR, Aganezov SS, Alekseyev MA, Allen JE, et al. Highly evolvable malaria vectors: The genomes of 16 Anopheles mosquitoes. Science. 2015;347:1258522.

12. Capella-Gutiérrez S, Silla-Martínez JM, Gabaldón T. trimAl: a tool for automated alignment trimming in large-scale phylogenetic analyses. Bioinformatics. 2009;25:1972–3.

13. Sánchez R, Serra F, Tárraga J, Medina I, Carbonell J, Pulido L, et al. Phylemon 2.0: a suite of web-tools for molecular evolution, phylogenetics, phylogenomics and hypotheses testing. Nucleic Acids Res. 2011;39:W470–4.

14. Rambaut A. FigTree [Internet]. 2018 [cited 2022 Jul 8]. Available from: http://tree.bio.ed.ac.uk/software/figtree/

15. Aho AV, Kernighan BW, Weinberger PJ. Awk — a pattern scanning and processing language. Softw Pract Exp. 1979;9:267–79.

16. Quinlan AR. BEDTools: The Swiss-Army Tool for Genome Feature Analysis. Curr Protoc Bioinforma. 2014;47:11.12.1-11.12.34.

17. RStudio Team. RStudio: Integrated Development Environment for R [Internet]. 2022. Available from: http://www.rstudio.com/

18. Liao Y, Smyth GK, Shi W. The R package Rsubread is easier, faster, cheaper and better for alignment and quantification of RNA sequencing reads. Nucleic Acids Res. 2019;47:e47.

19. Love MI, Huber W, Anders S. Moderated estimation of fold change and dispersion for RNA-seq data with DESeq2. Genome Biol. 2014;15:550.

20. Kolde R. pheatmap: Pretty Heatmaps [Internet]. 2019. Available from: https://CRAN.R-project.org/package=pheatmap
